# Efficacy and safety of normobaric hyperoxia for acute ischemic stroke: a systematic review and meta-analysis of randomized controlled trials

**DOI:** 10.1016/j.eclinm.2025.103701

**Published:** 2025-12-18

**Authors:** Qin Chen, Zhuoxi Wu, Feng Chen, Jingyun Wang, Xinming Ye, Hong Li

**Affiliations:** Department of Anesthesiology, Second Affiliated Hospital of Army Medical University, PLA, 83 Xinqiao Main Street, Shapingba District, Chongqing, 400037, China

**Keywords:** Normobaric hyperoxia, Acute ischemic stroke, Endovascular therapy, Meta-analysis

## Abstract

**Background:**

The neuroprotective effects of normobaric hyperoxia (NBHO) for treating acute ischemic stroke (AIS) remain unclear. This systematic review and meta-analysis evaluated the safety and functional outcomes of NBHO in AIS patients.

**Methods:**

We searched major databases until November 13, 2025, for randomized controlled trials (RCTs) comparing NBHO (≥2 h within 24 h of onset) with room air or low-flow oxygen in adult patients with AIS. Two reviewers independently screened studies, with disagreements resolved by a third reviewer. The primary outcome was functional independence (modified Rankin Scale [mRS] scores 0–2) at three months. Secondary outcomes included reduced disability (ordinal shift across mRS grades 0–6), early neurological recovery (changes in National Institutes of Health Stroke Scale [NIHSS] within 7 days), and infarct volume changes. Safety outcomes included 90-day mortality, symptomatic intracranial hemorrhage (sICH), and pneumonia. Outcomes eligible for meta-analysis were analyzed using a random-effects model (Paule-Mandel heterogeneity estimation) with Hartung-Knapp-Sidik-Jonkman (HKSJ) adjustment for the primary analysis and without HKSJ adjustment for secondary analysis. Registration: PROSPERO (CRD42024584308).

**Findings:**

Eight RCTs involving 804 participants were included. Six studies (n = 746) showed that NBHO improved functional independence (RR 1.28, HKSJ 95% CI 1.07–1.51; *P* = 0.015), reduced disability (cOR 1.72, HKSJ 95% CI 1.35–2.20; *P* = 0.002), and lowered mortality (RR 0.62, HKSJ 95% CI 0.39–0.99; *P* = 0.047) at three months. NBHO also decreased NIHSS scores at 72 h (MD –2.18, HKSJ 95% CI –3.45 to −0.90; *P* = 0.009) across five studies, though effects at other timepoints were significant only in secondary analysis. NBHO did not increase the risk of sICH (RR 0.79, HKSJ 95% CI 0.45–1.40; *P* = 0.347). Among patients receiving endovascular therapy (five studies), NBHO did not increase the risk of pneumonia (RR 0.97, HKSJ 95% CI 0.61–1.55; *P* = 0.863). Results for infarct volume and subgroup analyses were inconclusive due to limited data.

**Interpretation:**

In this meta-analysis of predominantly Chinese populations, NBHO may improve functional independence, reduce disability, and lower mortality at three months, and promote early neurological recovery at 72 h without compromising safety. Generalizability to other ethnic groups requires confirmation.

**Funding:**

This work is supported by the Cultivation Program of Clinical Research Special Project of The Second Affiliated Hospital of 10.13039/501100012397Army Medical University (Grant No. 2024F037).

**Research in context**

**Evidence before this study**

The neuroprotective effects of normobaric hyperoxia (NBHO) in acute ischemic stroke (AIS) remain controversial, and current guidelines advise against supplemental oxygen for non-hypoxic AIS patients. While early small randomized controlled trials (RCTs) showed transient or no sustained benefit, recent trials combining NBHO with endovascular therapy (EVT), such as OPENS-2, have suggested that it may improve patient outcomes. However, the evidence remains limited and inconsistent. To provide a current and comprehensive assessment of the efficacy and safety of NBHO, we conducted a systematic review and meta-analysis. We searched PubMed, Web of Science, Embase, the Cochrane Central Register of Controlled Trials, and ClinicalTrials.gov for RCTs published from database inception to November 13, 2025, using the terms “normobaric” AND (“oxygen” OR “hyperoxia”) AND “ischemic stroke”, without restrictions on language. After screening 2407 identified records, eight RCTs were included in the meta-analysis.

**Added value of this study**

This systematic review and meta-analysis provides a comprehensive evaluation of NBHO (≥2 h within 24 h of stroke onset) in AIS patients. The pooled data from six RCTs (n = 746), five of which were combined with EVT, demonstrated that NBHO was associated with improved functional independence (RR 1.28), reduced disability (cOR 1.72), and lower mortality (RR 0.62) at 90 days, without increasing the risk of symptomatic intracranial hemorrhage (sICH). Furthermore, an analysis of five trials indicated that NBHO promoted early neurological recovery, as shown by a reduction in National Institutes of Health Stroke Scale (NIHSS) scores at 72 h.

**Implications of all the available evidence**

This meta-analysis suggests that NBHO, particularly when combined with reperfusion therapy, is a promising, low-cost, and accessible neuroprotective strategy for patients with moderate to severe AIS. With a favorable safety profile and widespread availability, it can be implemented across diverse healthcare settings. Future trials should validate these findings in broader populations and across different reperfusion strategies, including non-Chinese cohorts and posterior circulation strokes, while also exploring optimal timing, duration, and patient selection for NBHO therapy.

## Introduction

Stroke is a leading cause of death and disability worldwide, with incidence rising sharply over the past decade.^1^ By 2050, it is projected to rank among the top three contributors to the global burden of disease, posing a critical concern for public health.^2^ Acute ischemic stroke (AIS) accounts for approximately 88% of all stroke cases.^3^ In AIS, timely revascularization through rapid restoration of cerebral blood flow can effectively rescue the ischemic penumbra, a region of functionally impaired but potentially viable tissue that typically surrounds an area of recent cerebral infarction, thereby improving neurological recovery.^4^^,^^5^ This is primarily achieved through reperfusion therapies for eligible patients, including the most broadly used intravenous thrombolysis (IVT) and the highly effective endovascular therapy (EVT), which achieves timely vascular recanalization and significantly improves functional recovery.^6^^,^^7^ However, due to delayed recanalization and extensive infarction, a majority of patients experience unfavorable neurological outcomes.^8^ Therefore, neuroprotective strategies that can extend the therapeutic window for reperfusion or preserve the ischemic penumbra hold great potential for advancing AIS management.

Normobaric hyperoxia (NBHO), a cost-effective, non-invasive, and convenient intervention, can be implemented in various healthcare settings and domiciliary environments, making it a promising neuroprotective strategy. NBHO involves delivering 40%–100% oxygen at normal atmospheric pressure via high-flow devices (typically 10–45 L/min; such as non-rebreather masks) in non-intubated patients, or via fraction of inspired oxygen (FiO_2_) titration in intubated patients.^9^ NBHO could improve neurological outcomes by increasing oxygen delivery to the ischemic penumbra, enhancing cerebral metabolism, preserving the blood–brain barrier, and thereby extending the therapeutic time window.^10^ However, it may be accompanied by the risks of vasoconstriction and pulmonary complications.^5^ Early exploratory trials on the neurological benefits of NBHO in AIS patients have reported inconsistent findings. Some trials showed that NBHO improved infarct evolution or functional recovery at various time points,^11^^,^^12^ while others observed no such therapeutic effects.^13^^,^^14^ Furthermore, an international multicenter RCT (N = 8003) found that low-flow oxygen did not improve functional outcomes in AIS patients.^15^ Given the limited and conflicting evidence, current guidelines do not recommend supplemental oxygen for non-hypoxic AIS patients.^7^^,^^16^ Recent landmark RCTs have shown that NBHO combined with EVT significantly reduced infarct progression and improved neurological recovery in AIS patients compared to low-flow oxygen therapy.^17^^,^^18^ These findings suggest that oxygen concentration may be a critical factor influencing neuroprotective effects. Therefore, a reassessment of NBHO within modern reperfusion frameworks is required. However, comprehensive meta-analyses assessing the efficacy and safety of NBHO in AIS patients are scarce. The conclusion from previous meta-analyses that oxygen therapy offered no neuroprotective benefits in AIS patients may be confounded by unstratified oxygen concentration and heterogeneous patient populations, including those not receiving reperfusion treatments such as EVT or thrombolysis.^19^^,^^20^

This study synthesized evidence from published RCTs to assess the efficacy of NBHO in patients with AIS, with the goal of informing optimal oxygen therapy strategies and refining clinical guidelines.

## Methods

### Registration of the systematic review and meta-analysis

This study was conducted in accordance with the Preferred Reporting Items for Systematic Reviews and Meta-Analyses (PRISMA), and the protocol was registered in PROSPERO on August 28, 2024 (registration ID: CRD42024584308).

### Search, eligibility criteria, and study selection

RCTs published up to August 28, 2024, were initially retrieved from PubMed, Web of Science, Embase, Cochrane Central Register of Controlled Trials, and ClinicalTrials.gov using predefined keywords and Boolean operators. An updated search was conducted on November 13, 2025, to identify eligible studies published after the initial search period. No language restrictions were applied. The search strategy is provided in Supplemental Table S1.

Inclusion criteria were based on the Population, Intervention, Comparison, Outcomes, and Study (PICOS) principle: (i) population: AIS patients aged 18 or above; (ii) intervention: NBHO was administered for at least 2 h within 24 h of stroke onset in AIS patients without other indications for supplemental oxygen therapy. This 2-h duration threshold was selected based on a previous dose-escalation study that identified it as the briefest tested exposure to show a trend in infarct volume reduction with safety, while efficacy became statistically significant at longer durations, therefore establishing it as a clinically practicable lower limit^21^; (iii) comparison: patients in the control group received room air or low-flow oxygen therapy (FiO_2_ < 40%, typically corresponding to 1–4 L/min delivered by nasal cannula or mask); (iv) outcomes: reported at least one of the following outcomes, including functional independence, disability reduction, early neurological recovery, infarct volume change, symptomatic intracranial hemorrhage (sICH), pneumonia, or 3-month mortality; (v) study design: RCT.

Exclusion criteria included conference abstracts, clinical protocols, systematic reviews, or meta-analyses, overlapping or duplicate reports, mixed stroke cohorts lacking isolated AIS data, or interventions not involving NBHO (confirmed by full text).

Following the predefined inclusion criteria, two reviewers (Q C and Z X W) independently screened titles and abstracts to identify potentially eligible RCTs. Full texts of selected studies were then assessed for final inclusion. References of included studies were also examined for additional eligible RCTs. Disagreements were resolved by consulting a third assessor (HL).

### Data extraction

Data from the included RCTs were recorded in a pre-designed table, including authors, title, journal, study design, year of publication, inclusion criteria, number of participants, treatments administered to the experimental and control groups, time from stroke onset to treatment, reperfusion strategies, outcome measures, and results. Two authors (Q C and Z X W) independently extracted the data, with any disagreements resolved through discussion. When original data were unavailable, the authors were contacted to obtain the necessary information.

### Primary and secondary outcomes

The primary outcome was functional independence, defined as a modified Rankin Scale (mRS) score of 0–2 at three months. Secondary outcomes included reduced disability, measured by the ordinal shift across mRS grades 0–6 at 90 days, early neurological recovery, defined by changes in the National Institute of Health Stroke Scale (NIHSS) from baseline within the first 7 days (4 h, 24 h, 72 h, and 7 days), and changes in infarct volume from baseline to 24–72 h after intervention. Safety outcomes included sICH within 24 h, pneumonia during hospitalization, and mortality at 3 months.

### Quality assessment and GRADE certainty assessment

Two reviewers (Q.C. and Z.X.W.) independently assessed the quality of the included RCTs using the revised Cochrane Risk of Bias 2.0 tool (RoB 2.0). Five domains were evaluated: randomization process, deviations from intended interventions, missing outcome data, outcome measurement, and selection of reported results. Each domain was rated as low risk of bias, some concerns, or high risk of bias.^22^ Disagreements were resolved by a third author (H.L). The certainty of evidence for each outcome was evaluated using the Grading of Recommendations, Assessment, Development, and Evaluation (GRADE) framework and classified as high, moderate, low, or very low.^23^

### Statistical analyses

Data analyses were performed using Stata 18 (Stata Corp) and R 4.3.2 with the meta package. Risk ratio (RR) with Hartung-Knapp-Sidik-Jonkman (HKSJ)-adjusted 95% confidence interval (CI) was used as the effect measure for dichotomous outcomes.^24^ Common odds ratio (cOR) with HKSJ-adjusted 95% CI was used as the effect measure for ordinal outcomes (shift across mRS grades). Mean difference (MD) or standardized mean difference (SMD) with HKSJ-adjusted 95% CI was used as the effect measure for continuous data. MD was used as the effect measure for outcomes measured on identical scales (such as NIHSS), whereas SMD was used as the effect measure for outcomes measured with heterogeneous instruments. However, uniform measurements throughout this analysis precluded the use of SMD. When studies reported continuous outcomes as medians with interquartile ranges or ranges, these values were converted to means and standard deviation (SD) using validated methods.^25^^,^^26^ Outcomes that were ineligible for meta-analysis due to non-normal distribution or heterogeneity in study design were summarized according to the Synthesis Without Meta-analysis (SWiM) reporting guideline.^27^ For outcomes eligible for meta-analysis, a random-effect model with Paule-Mandel estimate of the between-study variance and HKSJ-adjusted CI was systematically applied as the primary model for all outcomes with data from two or more studies.^28^ This approach specifically addressed inherent limitations, including small sample sizes, sparse outcome data, and methodological heterogeneity across trials, irrespective of statistical significance in formal heterogeneity testing. For the ordinal mRS shift outcome, a two-stage meta-analytic strategy was implemented. Study-specific cOR were first derived using proportional odds logistic regression, with the proportional odds assumption verified using Brant's test at a significance threshold of *P* > 0.10. Detected violations triggered sensitivity analyses using generalized ordered logit models.^29^ The resulting cOR estimates were subsequently pooled using Paule-Mandel between-study variance estimation and HKSJ 95% CI adjustment for a small number of studies. A preplanned secondary model using a random-effects model (random-effects with Paule-Mandel variance estimation but without HKSJ adjustment) was performed across all meta-analyzable outcomes to quantify model dependency. Given the potential imprecision of heterogeneity estimates in meta-analyses with limited studies, leave-one-out sensitivity analyses were conducted to identify sources of heterogeneity and assess the robustness of all meta-analyzable outcomes.^30^ Prespecified subgroup analyses were performed to explore potential sources of clinical heterogeneity and effect modification by stroke location (anterior circulation stroke [ACS] versus posterior circulation stroke [PCS]) and reperfusion strategy (EVT versus non-EVT). A two-sided *P* value < 0.05 was considered statistically significant. Heterogeneity among studies was assessed using I^2^ and Q statistic, with I^2^ > 50% indicating substantial heterogeneity across studies. Publication bias was not assessed when fewer than 10 studies were included.

### Ethics statement

No ethical approval was required for this study, as it was based solely on publicly available, aggregated data.

### Role of the funding source

The funder of this study, the Cultivation Program of Clinical Research Special Project of The Second Affiliated Hospital of Army Medical University (Grant No. 2024F037), had no role in study design, data collection, data analysis, data interpretation, or writing of the report.

## Results

### Study selection and study characteristics

**Fig. 1:**
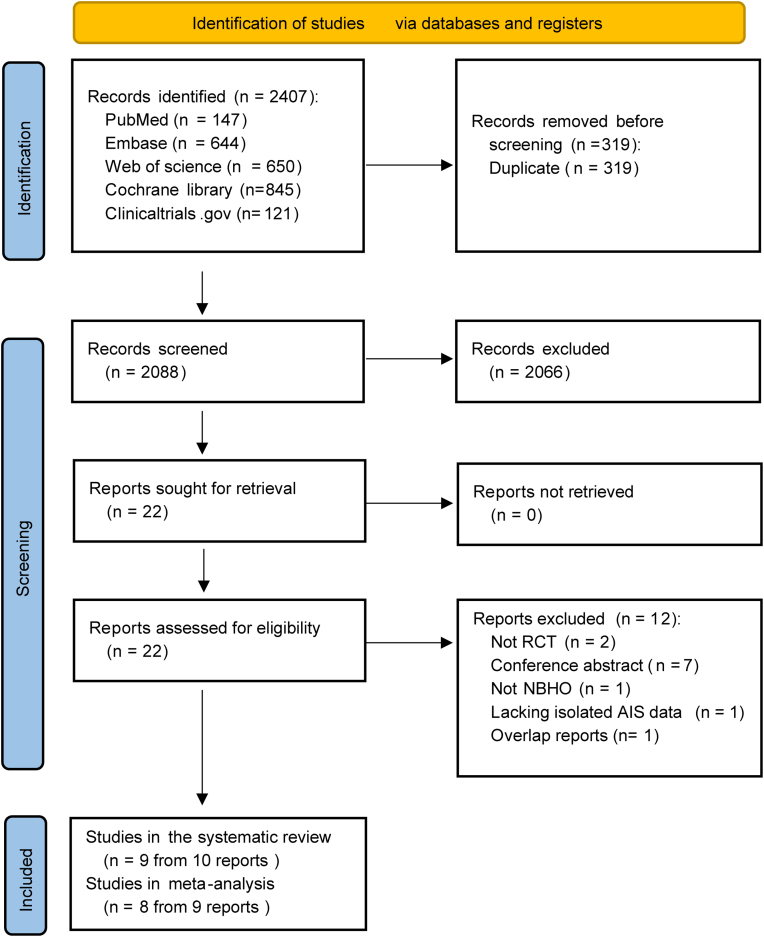
Flowchart of study selection.

A total of 2407 relevant studies were initially retrieved from these databases. After removing duplicates, 2088 studies remained. Screening of titles and abstracts excluded 2066 studies, leaving 22 publications for full-text review. Among these, three publications were based on the same cohort.^11^^,^^31^^,^^32^ Two^11^^,^^32^ of these were retained as they reported eligible primary and secondary outcomes. One study was excluded because the title and abstract did not report the oxygen concentration, and a full-text review confirmed that it did not involve NBHO therapy.^33^ Another study was excluded because NBHO outcomes for AIS patients could not be extracted from mixed AIS and hemorrhagic stroke cohorts.^34^ The trial NCT00414726^35^ was excluded from the primary analysis due to concerns over baseline imbalances that compromised its internal validity. This concern was acknowledged by the principal investigator, who stated that the NBHO group had more severe strokes and pre-existing illnesses.^14^^,^^36^ Sensitivity analyses incorporating this trial are presented in Supplementary File 1, along with its standalone risk of bias assessment (judged as high risk). Ultimately, eight independent RCTs involving 804 AIS patients were included in the meta-analysis.^11^, ^12^, ^13^^,^^17^^,^^18^^,^^21^^,^^37^^,^^38^ The study screening process is illustrated in Fig. 1. The main characteristics of these eligible RCTs are summarized in Table 1. One study^21^ contained three NBHO subgroups with different treatment durations (2 h, 4 h, and 6 h; n = 25 per subgroup). To avoid unit-of-analysis errors, these subgroups were integrated into a single intervention group (n = 75) and compared with a shared control group (n = 25). Seven RCTs enrolled ACS patients, and one targeted PCS. The initiation time of the intervention ranged from 4.5 to 24 h after AIS onset. Five RCTs combined NBHO with EVT. Follow-up was 90 days in seven studies and seven days in one study. The proportion of male patients ranged from 47.6% to 84.1%, and the mean age varied from 50.6 to 71.4 years. Baseline NIHSS scores yielded median values of 13–22.5 and mean values of 12–14. The NBHO oxygen regimens ranged from 10 to 45 L/min with FiO_2_ 50%–100% via facemask for 2–8 h following randomization.

**Table 1:** Characteristics of the trials.

Author/year	Country	Patient characteristics	Intervention (NBHO) versus control (C)	N	Men (%)	Age (years)^a^	Baseline NIHSS^b^	EVT	Outcomes
Singhal 2005	America	Anterior AIS ≤15 h	NBHO: 45 L/min for 8 h	9	44	65.0 ± 17.1	14 (4–22)	NO	mRS; NIHSS; Lesion volume
			C: room air 1–3 L/min	7	43	71.4 ± 17.6	11 (8–21)		
Padma 2010	India	Anterior AIS ≤12 h	NBHO: 10 L/min for 12 h	20	NA	NA	14.25 ± 3.85	NO	mRS; NIHSS; Lesion volume
			C: room air/oxygen 2 L/min	20	NA	NA	12.7 ± 4.52		
Shi 2017	China	Anterior AIS ≤4.5 h	NBHO:10 L/min for 4 h	9	66.7	64.6 ± 11	12 ± 4.59	NO	NIHSS;
			C: room air	9	71.4	50.6 ± 7.25	12.3 ± 3.96		
Cheng 2021	China	Anterior AIS ≤6 h	NBHO: 15 L/min for 6 h	88	63.6	63.8 ± 11.5	17 (14–18)	YES	mRS; NIHSS; Lesion volume; mortality sICH; pneumonia
			C: oxygen 3 L/min	87	58.6	65.9 ± 10.5	16 (14–19)		
Cheng 2022	China	Posterior AIS ≤6 h	NBHO: 15 L/min for 6 h	44	84.1	64.02 ± 11.44	22.5 (13–26)	YES	mRS; NIHSS; Lesion volume; mortality sICH; pneumonia
			C: oxygen 3 L/min	43	74.4	63.53 ± 11.22	21 (18–27)		
Li 2022	China	Anterior AIS ≤6 h	NBHO: 10 L/min for 4 h	43	31	62.0 ± 11.7	14 (12–17)	YES	mRS; NIHSS; Lesion volume; mortality sICH; pneumonia
			C: room air	43	29	64.0 ± 9.9	13 (12–16)		
Li 2024	China	Anterior AIS ≤24 h	NBHO: 2 h (10 L/min), 4 h (10 L/min), 6 h (10 L/min)	75 (25 per group)	69.3	61.9 ± 8.6	10 (8–13)	YES	mRS; NIHSS; Lesion volume; mortality; sICH; pneumonia
			C: 1 L/min oxygen for 4 h	25	52	63.9 ± 9.6	11 (7–14)		
Li 2025	China	Anterior AIS ≤6 h	NBHO: 10 L/min for 4 h	140	75	63.9 ± 9.7	14 (12–16.2)	YES	mRS; NIHSS; Lesion volume; mortality; sICH; pneumonia
			C: oxygen 3 L/min or FiO2 0.3	142	72	64.9 ± 11.2	14 (12–16.8)		

Abbreviations: NBHO, normobaric hyperoxia; C, control; AIS, acute ischemic stroke; EVT, endovascular therapy; mRS, modified Rankin Scale; NIHSS, National Institutes of Health Stroke scale; sICH, symptomatic intracranial hemorrhage; IQR, interquartile range; SD, standard deviation; NA, not available.

a Age (years) data format: mean ± SD.

b Baseline NIHSS data format: mean (SD) or median (IQR).

### Quality assessment of risk of bias and quality of evidence

The RoB 2.0 tool was used to assess the risk of bias for the included studies. Among the 8 studies, 4 were judged to have some concerns^12^^,^^13^^,^^37^^,^^38^ and 4 were rated as^11^^,^^17^^,^^18^^,^^21^ having a low risk of bias (Supplemental Figures S1 and S2). According to the GRADE assessment, the certainty of evidence was very low for all outcomes except reduced disability (rated as low) (Supplemental Table S2).

### Efficacy outcomes

#### Functional independence

**Fig. 2:**
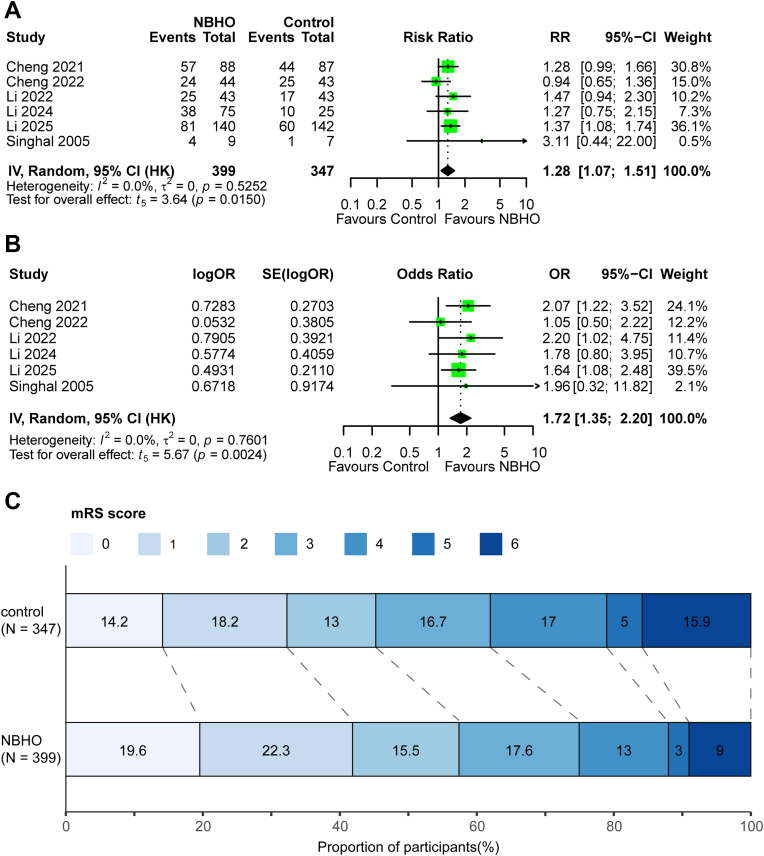
Forest plot of functional outcomes: (A) independence (mRS 0–2 at 90 days), (B) reduced disability (ordinal shift across mRS grades 0–6 at 90 days), and (C) distribution of modified Rankin Scale scores at 90 days. Note: HK, Hartung-Knapp-Sidik-Jonkman adjusted random-effects model (primary model); mRs, modified Rankin Scale score, ranging from 0 to 6, where 0 indicates no symptoms, 1 no clinically significant disability, 2 slight disability, 3 moderate disability, 4 moderately severe disability, 5 severe disability, and 6 death. The numbers in the bars represent the proportions of patients at each score; CI, confidence interval; RR, risk ratio; OR, common odds ratio (applied for reduced disability); NBHO, Normobaric hyperoxia. The definitions and model specifications above apply uniformly to all forest plots presented in this manuscript.

Seven studies assessed functional ability using the mRS at 3 months after stroke. Among them, six studies reported the distribution of mRS scores. In these studies, the proportion of patients achieving functional independence at three months was higher in the NBHO group (57.32%, 95% CI 52.39%–62.11%) than in the control group (45.41%, 95% CI 37.81%–53.23%). The pooled RR was 1.28 (HKSJ 95% CI 1.07–1.51; *P* = 0.015; I^2^ = 0%; Fig. 2A) in the primary model and 1.28 (95% CI 1.10–1.47; *P* < 0.001; I^2^ = 0%; Supplemental Figure S3A) in the secondary model. Sensitivity analysis (leave one out) revealed model-dependent robustness: exclusion of the study Cheng 2021 and Li 2025 abolished significance in the primary model despite low heterogeneity (Supplemental Figure S4A), whereas the secondary model remained robust across all exclusions (Supplemental Figure S5A). Subgroup analyses were conducted for the ACS and EVT cohorts, revealing benefits with pooled RRs of 1.35 (*P* < 0.002) and 1.27 (*P* < 0.003), respectively, in both the primary and secondary models. The results are shown in Supplemental Figures S6A, B and S7A, B. However, formal meta-analysis was not feasible for the PCS and non-EVT subgroups, as each included only one study.

#### Reduced disability

Six studies reported reduced disability. Both primary and secondary models showed significantly better outcomes with NBHO therapy. The primary model yielded a cOR 1.72 (HKSJ 95% CI, 1.35–2.20; *P* = 0.002; I^2^ = 0%; Fig. 2B; Fig. 2C), and the secondary model reported a cOR 1.72 (95% CI, 1.33–2.23; *P* < 0.0001; I^2^ = 0%; Supplemental Figure S3B). The proportional odds assumption was validated (Brant test *P* = 0.415). Sensitivity analysis (leave one out) showed robustness in both models (Supplemental Figures S4B and S5B). Subgroup analyses were conducted for the ACS and EVT cohorts, revealing benefits with cOR of 1.84 (*P* < 0.001) and 1.72 (*P* < 0.001), respectively, in both models (Supplemental Figures S6C, D and S7C, D). Formal meta-analysis was not feasible for the PCS and non-EVT subgroups, as each included only one study.

#### Early neurological recovery

Eight studies reported early neurological recovery based on changes in NIHSS scores within the first seven days. NIHSS assessments were performed at 4 h (two studies), 24 h (eight studies), 72 h (five studies), and seven days (six studies).

**Fig. 3:**
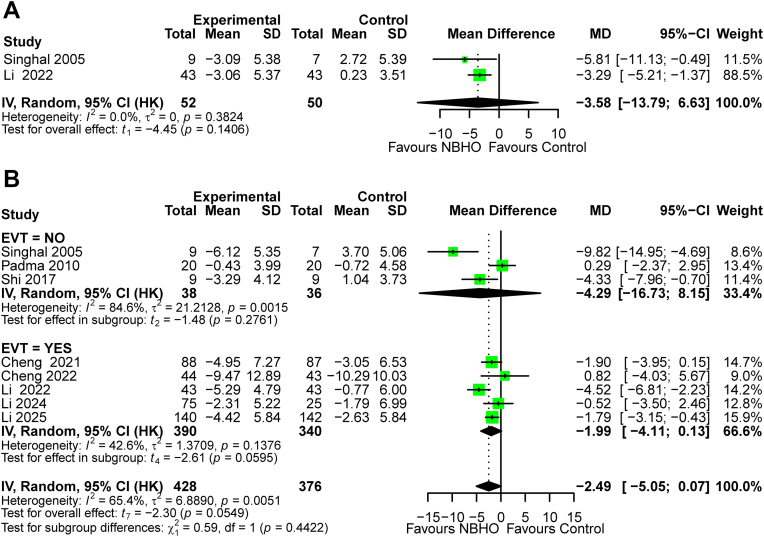
Forest plots of NIHSS score changes: (A) 4 h and (B) 24 h. Note: MD, Mean difference; SD, standard deviation; NIHSS, National Institutes of Health Stroke Scale; EVT, endovascular therapy. The definitions and model specifications above apply uniformly to all forest plots presented in this manuscript.

At 4 h, the primary model showed no significant differences in changes in NIHSS scores between NBHO and control groups (MD −3.58, HKSJ 95% CI −13.79 to 6.63; *P* = 0.141; I^2^ = 0%; Fig. 3A). In contrast, the secondary model indicated a significant improvement with NBHO (MD −3.58, 95% CI −5.38 to −1.78, *P* < 0.001; I^2^ = 0%; Supplemental Figure S9A). A leave-one-out sensitivity analysis was not performed as only two studies were included.

At 24 h, the primary model did not demonstrate a significant effect (MD −2.49, HKSJ 95% CI −5.05 to 0.07, *P* = 0.055, I^2^ = 65.4%; Fig. 3B). In contrast, the secondary model indicated a significant reduction in NIHSS score (MD −2.49, 95% CI −4.62 to −0.37, *P* = 0.021, I^2^ = 65.4%; Supplemental Figure S10A). No significant effect modification by EVT status was detected (*P* for subgroup difference = 0.442 in both models), despite isolated significance in the EVT subgroup of the secondary model. Given that only one study included PCS patients, no subgroup analysis was conducted for PCS. In the ACS subgroup, NBHO was associated with significantly lower NIHSS scores in both models (primary model: MD −2.83, HKSJ 95% CI −5.63 to −0.02, *P* = 0.049, I^2^ = 68%; Supplemental Figure S8; secondary model: MD −2.83, 95% CI −5.07 to −0.58, *P* = 0.0014, I^2^ = 68%; Supplemental Figure S9B). Sensitivity analyses (leave-one-out) indicated fragility in both models (Supplemental Figure S11A and S12A).

**Fig. 4:**
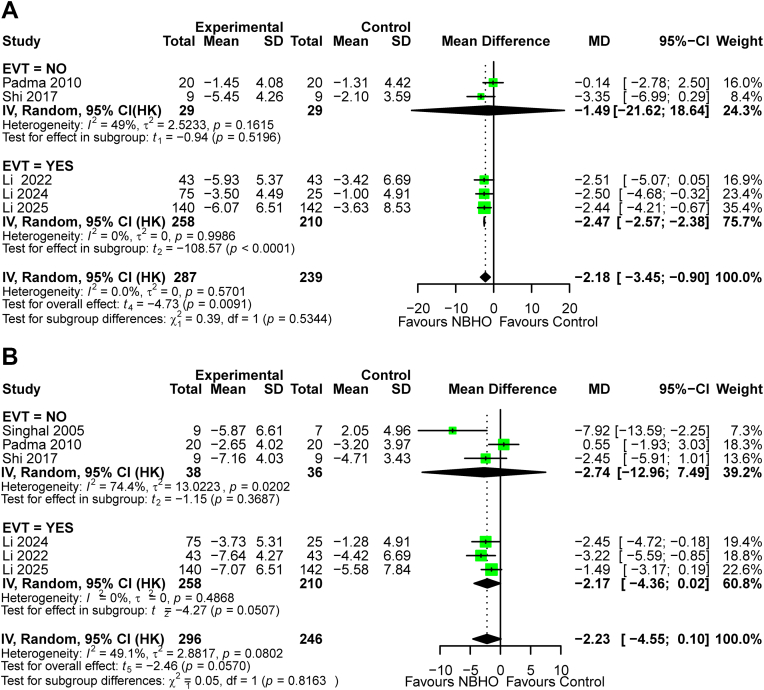
Forest plots of NIHSS score changes: (A) 72 h and (B) 7 days.

At 72 h, NBHO showed significant overall benefit in both models (primary model: MD −2.18, HKSJ 95% CI −3.45 to −0.90, *P* = 0.009, I^2^ = 0%; Fig. 4A; secondary model: MD −2.18, 95% CI −3.23 to −1.12, *P* < 0.001, I^2^ = 0%; Supplemental Figure S10B). No significant effect modification by EVT status was observed (primary model: *P* for subgroup difference = 0.534; secondary model: *P* for subgroup difference = 0.563), although isolated significance was noted in the EVT subgroups of both models. Sensitivity analyses showed (leave-one-out) fragility in the primary model (Supplemental Figure S11B), but robustness in the secondary model (Supplemental Figure S12B).

At seven days, outcomes were non-significant overall and across all subgroups in the primary model (MD −2.23, HKSJ 95% CI −4.55 to 0.1; *P* = 0.057; I^2^ = 49.1%; Fig. 4B), contrasting with a significant reduction of NIHSS score in the secondary model (MD −2.23, 95% CI −4.00 to −0.45; *P* = 0.014; I^2^ = 0%; Supplemental Figure S10C). No significant effect modification by EVT status was observed (primary model: *P* for subgroup difference = 0.816; secondary model: *P* for subgroup difference = 0.818), despite isolated significance in the EVT subgroups of both models. Sensitivity analyses (leave-one-out) showed fragility in both models (Supplemental Figures S11C and S12C).

#### Changes in infarct volume

The prespecified outcome of infarct volume changes (from baseline to 24–72 h post-intervention) showed substantial heterogeneity across studies. This variation was primarily due to non-normally distributed data, limited baseline volumetric characterization (only two studies reported it), and inconsistencies in the timing of outcome assessments, which ranged from 4 h to 72 h. Given these limitations, the analysis was limited to reporting absolute infarct volume at each evaluation time point (baseline data excluded) according to the SWiM guideline. Vote counting based on effect direction revealed that four RCTs^11^^,^^17^^,^^18^^,^^21^^,^^38^ reported smaller infarct volumes in the NBHO group compared with controls at their respective time points (24–72 h), whereas three RCTs^11^^,^^13^^,^^37^ showed no significant differences between groups (Supplemental Table S3).

### Safety outcomes

#### 90-Day mortality

**Fig. 5:**
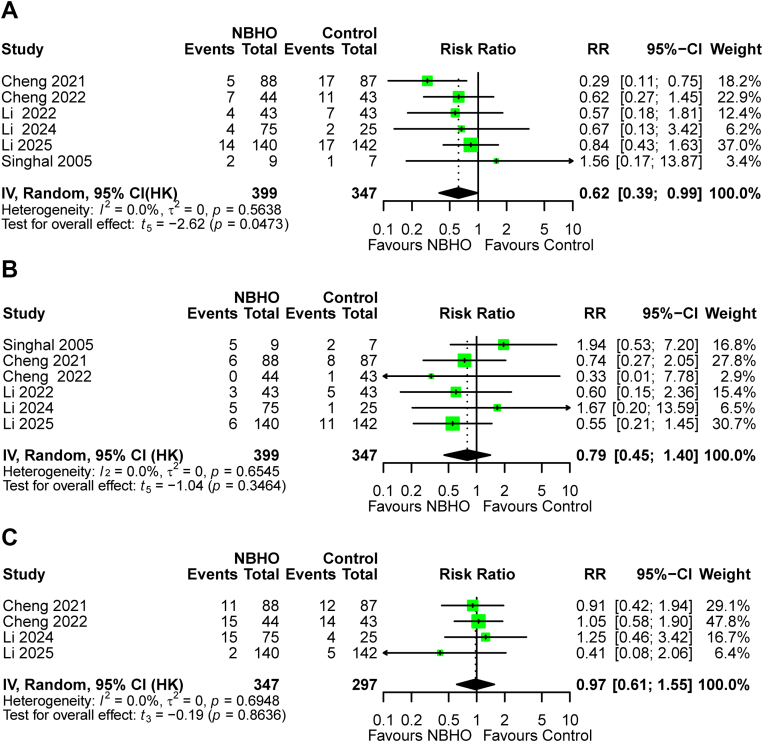
Forest plots of safety outcomes: (A) 90-day mortality, (B) 24-h sICH, and (C) pneumonia.

Six studies involving 746 participants reported data on 90-day mortality. The NBHO group had a mortality rate of 9.63% (95% CI 6.50%–14.06%) compared with 16.55% (95% CI 12.39%–21.76%) in the control group. The primary model yielded a significant risk reduction (RR 0.62, HKSJ 95% CI 0.39–0.99; *P* = 0.047; I^2^ = 0%; Fig. 5A). Similarly, the secondary model indicated that NBHO significantly reduced mortality (RR 0.62, 95% CI 0.41–0.93; *P* = 0.021; I^2^ = 0%; Supplemental Figure 13A). Sensitivity analyses (leave one out) demonstrated a lack of robustness in both models (Supplemental Figures S14A and S15A). The model-dependent divergence was observed in the ACS and EVT subgroups: non-significant in the primary model (Supplemental Figure S16A and B) versus significant in the secondary model (Supplemental Figure S17A and B).

#### Symptomatic intracranial hemorrhage

Six studies involving 746 participants reported data on sICH. Overall, 8.21% (95% CI 2.81%–21.68%) of patients in the NBHO group and 8.93% (95% CI 5.41%–14.40%) in the control group experienced sICH. Both models showed no significant difference between the two groups (primary model: RR 0.79, HKSJ 95% CI 0.45–1.40, *P* = 0.346, I^2^ = 0%; Fig. 5B; secondary model: RR 0.79, 95% CI 0.46–1.36; *P* = 0.399; I^2^ = 0%; Supplemental Figure S13B), with robust leave-one-out sensitivity analyses (Supplemental Figures S14B and S15B) and consistent non-significant effects across subgroups (Supplemental Figures S16C, D and S17C, D).

#### Pneumonia

Four studies involving 644 participants reported data on pneumonia. The incidence rates were similar between the NBHO group (12.30%, 95% CI 3.17%–37.56%) and the control group (13.48%, 95% CI 5.18%–30.79%). Both models suggested no significant difference between the two groups (primary model: RR 0.97, HKSJ 95% CI 0.61–1.55; *P* = 0.864; I^2^ = 0%; Fig. 5C; secondary model: RR 0.97, 95% CI 0.65–1.47; *P* = 0.897; I^2^ = 0%; Supplemental Figure S13C), with robust leave one out sensitivity analyses (Supplemental Figures S14C and S15C) and consistent non-significant effects across subgroups (Supplemental Figures S16E and S17E).

## Discussion

This meta-analysis indicates that NBHO improved functional independence, reduced disability, and lowered mortality at three months. Moreover, it improved neurological recovery at 72 h, with nominally significant effects at 4 h, 24 h, and 7 days observed only in secondary model. Additionally, it did not increase the risk of sICH, nor did it increase the risk of pneumonia in patients receiving EVT. Prespecified subgroups (ACS or EVT) showed no significant effect modifications. These findings suggest the potential clinical utility of NBHO with acceptable safety. However, they should be interpreted cautiously due to sensitivity fragility, a limited number of studies, unquantified publication bias, and overall evidence certainty ranging from very low to low, underscoring the need for confirmatory trials.

This meta-analysis evaluates the therapeutic effects of NBHO in individuals with AIS. Our results differ from previous meta-analyses reporting a neutral neuroprotective effect of oxygen therapy in AIS.^19^^,^^20^ The divergence may be largely attributed to fundamental differences in intervention protocols and population characteristics. Earlier studies primarily assessed routine oxygen regimens (<2% of patients with NBHO exposure) without concurrent revascularization in moderate strokes (median NIHSS 5–7) and with delayed initiation (median 19–20 h post-onset). In contrast, our analysis focused on a targeted NBHO strategy, with 89.7% of cases receiving EVT for moderate-severe strokes (median NIHSS 13–22.5), and 80% of patients initiating treatment within the critical 6-h window—a timeframe when more than 80% of the penumbra remains salvageable.^4^ A post hoc analysis of the OPENS-1 trial revealed enhanced NBHO efficacy with superior outcomes in patients treated within 6 h of onset and significantly reduced infarct volumes in those with baseline NIHSS scores ≥13 compared to the 6–12 subgroup.^18^^,^^39^ These findings collectively suggest that earlier initiation of combined NBHO and EVT therapy in moderate-severe AIS may account for these differences in outcomes.

Model-dependent variations in early neurological recovery (4 h/24 h/7 d) were observed. The HKSJ model was selected for the primary analysis because it can provide a wider 95%CI and reduce the risk of type I error, highlighting the sparsity of evidence. Substantial heterogeneity was observed at 24 h and 7 days. Predefined subgroup analyses failed to explain this heterogeneity. Potential contributors included stroke location, revascularization approaches, oxygen delivery methods, stroke severity, and time from stroke onset to NBHO initiation. Given these limitations and the absence of significant subgroup interactions, definitive conclusions regarding NBHO efficacy in ACS/EVT cohorts cannot be drawn despite favorable point estimates. Although the changes in NIHSS scores were statistically significant at 72 h, the absence of an established minimal clinically important difference (MCID) limits clinical interpretation. Heterogeneous MCID definitions (2–10 points) across trials stemmed from variations in study protocols and populations,^40^^,^^41^ with two trials in our meta-analysis defining a 4-point reduction threshold.^17^^,^^18^ This necessitated the use of an estimated threshold (0.5 × pooled SD) to evaluate the precision of effect estimates using CIs in GRADE evidence quality assessment.^42^ The clinical impact of NBHO on early neurological recovery in AIS patients remains to be clarified.

Four of seven RCTs reported that NBHO reduced infarct volumes within 24–72 h post-stroke. One trial that reported neutral findings may have been affected by a high dropout rate during the 24-h MRI follow-up. In another RCT, the therapeutic effect of NBHO in PCS was not statistically significant, in contrast to the benefits reported in ACS using the same NBHO protocol by the same research team. This discrepancy may be attributed to the larger ischemic penumbra typically observed in ACS,^43^ whereas PCS involves regions with higher neuronal density and critical brainstem functional zones.^44^ Notably, when combined with EVT,^21^ NBHO showed more pronounced benefits sustained up to 72 h, whereas NBHO alone yielded only transient infarct volume reduction within the first 4 h, with effects diminishing by 24 h.^11^ These findings suggest that NBHO may improve AIS outcomes by reducing infarct volume expansion. Our findings align with previous literature,^9^ indicating NBHO alone may not significantly improve AIS prognosis, potentially due to ongoing infarct growth in the absence of reperfusion. However, when combined with reperfusion strategies, NBHO may improve neurological outcomes. Reducing infarct volume not only alleviates neurological deficits but also may decrease the risk of long-term disability, thereby improving patients’ quality of life.

When evaluating NBHO for AIS treatment, both therapeutic efficacy and safety profile must be considered. Pooled safety data showed no increased risk of sICH or pneumonia with NBHO, while revealing a reduction in mortality. A previous study NCT00414726^35^ associated hyperoxia-related harm with high flow rates (such as 45 L/min) and long durations (such as ≥8 h). However, the investigators attributed the observed mortality imbalance to baseline severity disparities, rather than NBHO therapy itself.^14^^,^^36^ Among the studies included in our safety analysis, only one small-scale trial exceeded these thresholds, representing just 2% of the total sample size. Importantly, this trial reported neuroprotective effects of NBHO without significant safety differences compared with controls. Nevertheless, the data specifically addressing safety under these extreme parameters remain very limited. Therefore, as a precautionary measure until more robust evidence is available, it is prudent to avoid unnecessarily prolonged exposure to very high-flow NBHO.

This study has several limitations. First, the small number of included studies precluded assessment of publication bias, which could overestimate the treatment effects of NBHO if negative or null studies remained unpublished and prevented evaluation of subgroup factor interactions. Sensitivity analyses indicated statistically fragile estimates, potentially limiting generalizability to comparable cohorts. Second, as our findings were primarily derived from two Chinese research teams focusing on anterior circulation EVT in a predominantly atherosclerotic stroke population, which differed from the cardioembolic-dominant etiology typical in Western cohorts, and further confined by the exclusion of the non-Chinese NCT00414726 trial, extrapolation may be particularly limited for non-Chinese cohorts, patients with posterior circulation stroke, and other healthcare institutions. Third, quantitative meta-analysis was not performed because infarct volume data were abnormally distributed, potentially reducing the precision and comparability of these outcomes. Lastly, the lack of a defined NIHSS MCID necessitated reliance on an estimated threshold, limiting the precision of assessing the clinical effects of NBHO on early functional recovery in AIS patients.

Future multicenter RCTs should validate the effects of NBHO across relevant subgroups (such as stroke location, treatment timing) and integrate advanced imaging biomarkers to refine precision therapy strategies, thereby enabling precision neuroprotection through individualized therapeutic algorithms in ischemic stroke. Additionally, prospective cohort studies should establish NIHSS MCID using anchor-based methods to assess clinical effects. The results of several large-scale, multicenter RCTs (such as NCT05965687, NCT05965193, and NCT06224426) that further investigate the neuroprotective effect of NBHO combined with reperfusion therapy on outcomes in AIS patients are being closely monitored.

In conclusion, this study indicates that NBHO may improve functional independence, reduce disability, and lower mortality at three months, and promote early neurological recovery at 72 h. It is not associated with an increased risk of sICH in the AIS population or of pneumonia in EVT-treated patients. These findings should be interpreted cautiously due to model-dependent robustness in sensitivity testing and the limited number of trials. Furthermore, as the current evidence is predominantly derived from Chinese populations, the generalizability of these results should be confirmed in broader, multi-ethnic cohorts.

## Contributors

Qin Chen and Zhuoxi Wu conceived and designed the study, searched the literature, extracted the data, and reviewed available studies. Qin Chen and Zhuoxi Wu accessed and verified the underlying data. Qin Chen and Feng Chen analyzed the results and co-wrote the paper. Jingyun Wang and Xinming Ye reviewed the paper. Hong Li obtained the funding and revised the manuscript. All the authors reviewed and approved the final manuscript, confirmed full access to all study data, and accept responsibility for its submission for publication.

## Data sharing statement

The datasets used and analyzed during the current study are available from the corresponding author on reasonable request.

## Declaration of interests

We declare no competing interests.
