# Supplementary material for: Efficacy and safety of normobaric hyperoxia for acute ischemic stroke: a systematic review and meta-analysis of randomized controlled trials
Source: eClinicalMedicine. 2025 Dec 18;91:103701. doi: 10.1016/j.eclinm.2025.103701 (PMC12775875; doi:10.1016/j.eclinm.2025.103701)
Supplement: Supplemental Materials [file mmc1.docx]

**Efficacy and safety of normobaric hyperoxia for acute ischemic stroke: a systematic review and meta-analysis of randomized controlled trials**

**Supplemental Content Appendix**

**Content**

1. **Supplemental Table S1** Search strategy.

2. **Supplemental Figure S1** Quality assessment of included studies using the Cochrane Risk of Bias 2.0 tool.

3. **Supplemental Figure S2** Risk of bias plot over all studies.

4. **Supplemental Table S2** GRADE Level of evidence.

5. **Supplemental Figure S3** Forest plot of functional outcomes by secondary model:(A) independence (mRS 0-2 at 90 days) and (B) reduced disability (ordinal shift across mRS grades 0-6 at 90 days).

6.**Supplemental Figure S4** Sensitivity analysis of functional outcomes by primary model: (A) independence (mRS 0-2 at 90 days) and (B) reduced disability (ordinal shift across mRS grades 0-6 at 90 days).

7.**Supplemental Figure S5** Sensitivity analysis of functional outcomes by secondary model: (A) independence (mRS 0-2 at 90 days) and (B) reduced disability (ordinal shift across mRS grades 0-6 at 90 days).

8. **Supplemental Figure S6** Forest plots of subgroup analysis for functional outcomes by primary model: (A) independence (mRS 0-2 at 90 days) in anterior circulation stroke subgroup, (B) independence (mRS 0-2 at 90 days) in EVT subgroup (C) reduced disability (ordinal shift across mRS grades 0-6 at 90 days) in anterior circulation stroke subgroup, and (D) reduced disability (ordinal shift across mRS grades 0-6 at 90 days) in EVT subgroup.

9. **Supplemental Figure S7** Forest plots of subgroup analysis for functional outcomes by secondary model: (A) independence (mRS 0-2 at 90 days) in anterior circulation stroke subgroup, (B) independence (mRS 0-2 at 90 days) in EVT subgroup (C) reduced disability (ordinal shift across mRS grades 0-6 at 90 days) in anterior circulation stroke subgroup, and (D) reduced disability (ordinal shift across mRS grades 0-6 at 90 days) in EVT subgroup.

10. **Supplemental Figure S8** Forest plots of NIHSS scores changes by primary model: at 24 hours in anterior subgroup.

11. **Supplemental Figure S9** Forest plots of efficacy outcomes by secondary model: (A) NIHSS scores changes at 4 hours and (B) NIHSS scores changes at 24 hours in anterior subgroup.

12. **Supplemental Figure S10** Forest plots of NIHSS scores changes by secondary model: (A) 24 hours, (B) 72 hours, and (C) 7 days.

13. **Supplemental Figure S11** Sensitivity analysis of NIHSS scores changes by primary model: (A) 24 hours, (B) 72 hours, and (C) 7 days.

14. **Supplemental Figure S12** Sensitivity analysis of NIHSS scores changes by secondary model: (A) 24 hours, (B) 72 hours, and (C) 7 days.

15. **Supplemental Table S3** Infarct volume across studies and time points.

16. **Supplemental Figure S13** Forest plots of safety outcomes by secondary model: (A) 90-day mortality, (B) 24-hour sICH, and (C) pneumonia.

17.**Supplemental Figure S14** Sensitivity analysis of safety outcomes by primary model: (A) 90-day mortality, (B) 24-hour sICH, and (C) pneumonia.

18.**Supplemental Figure S15** Sensitivity analysis of safety outcomes by secondary model: (A) 90-day mortality, (B) 24-hour sICH, and (C) pneumonia.

19. **Supplemental Figure S16** Forest plots of subgroup analysis for safety outcomes by primary model: (A) 90-day mortality in anterior subgroup, (B) 90-day mortality in EVT subgroup, (C) 24-hour sICH in anterior subgroup, (D) 24-hour sICH in EVT subgroup, and (E) pneumonia in anterior subgroup.

20.**Supplemental Figure S17** Forest plots of subgroup analysis for safety outcomes by secondary model: (A) 90-day mortality in anterior subgroup, (B) 90-day mortality in EVT subgroup, (C) 24-hour sICH in anterior subgroup, (D) 24-hour sICH in EVT subgroup, and (E) pneumonia in anterior subgroup.

1. **Supplemental Table S1** Search strategy

| Database | Search number | | Search strategy | Results |
| --- | --- | --- | --- | --- |
| Pubmed | #1 | "Ischemic Stroke"[Mesh] | | 19079 |
|  | #2 | "Ischemic Stroke*"[Title/Abstract] OR "Ischaemic Stroke*"[Title/Abstract] OR "Cryptogenic Embolism Stroke*"[Title/Abstract] OR "Cryptogenic Stroke*"[Title/Abstract] OR "Wake up Stroke*"[Title/Abstract] OR "acute stroke*"[Title/Abstract] | | 107142 |
|  | #3 | "Oxygen"[Mesh] OR "Oxygen Inhalation Therapy"[Mesh] OR "hyperoxia"[Mesh] | | 236046 |
|  | #4 | "Oxygen"[Title/Abstract] OR "oxigen"[Title/Abstract] OR "dioxygen"[Title/Abstract] OR "O2"[Title/Abstract] OR "hyperoxia"[Title/Abstract] | | 770716 |
|  | #5 | "inspir*"[Title/Abstract] OR "inhal*"[Title/Abstract] OR "fraction*"[Title/Abstract] OR "concentrat*"[Title/Abstract] OR "suppl*"[Title/Abstract] OR "therap*"[Title/Abstract] OR "administr*"[Title/Abstract] OR "dosag*"[Title/Abstract] OR "dose*"[Title/Abstract] OR "dosing*"[Title/Abstract] OR "normobaric"[Title/Abstract] OR "eubaric"[Title/Abstract] | | 9287405 |
|  | #6 | ((randomized controlled trial[pt] OR controlled clinical trial[pt] OR randomized[tiab] OR placebo[tiab] OR clinical trials as topic[mesh:noexp] OR randomly[tiab] OR trial[ti]) NOT (animals [mh] NOT (humans [mh] AND animals[mh]))) NOT ("comment" OR "editorial"[pt] OR "interview"[pt] OR "letter"[pt] OR "news"[pt]) | | 1554244 |
|  | #7 | (#1 OR #2) AND (#5 AND #4 OR #3) AND #6 | | 147 |
| Embase | #1 | 'ischemic stroke'/exp OR 'ischemic stroke' | | 151367 |
|  | #2 | ('ischemic stroke*' OR 'ischaemic stroke*' OR 'cryptogenic embolism stroke*' OR 'cryptogenic stroke*' OR 'wake up stroke*' OR 'acute stroke*'): ti,ab,kw | | 183494 |
|  | #3 | ('oxygen therapy'/exp OR 'oxygen'/exp OR 'hyperoxia'/exp | | 428690 |
|  | #4 | 'oxygen' OR 'oxigen' OR 'dioxygen' OR 'O2' OR 'hyperoxia'): ti,ab,kw | | 892618 |
|  | #5 | ('inspir*' OR 'inhal*' OR 'fraction*' OR 'concentrat*' OR 'suppl*' OR 'therap*' OR 'administr*' OR 'dosag*' OR 'dose*' OR 'dosing*' OR 'normobaric' OR 'eubaric'): ti,ab,kw | | 12654486 |
|  | #6 | 'crossover procedure':de OR 'double-blind procedure':de OR 'randomized controlled trial':de OR 'single-blind procedure':de OR random*:de,ab,ti OR factorial*:de,ab,ti OR crossover*:de,ab,ti OR ((cross NEXT/1 over*):de,ab,ti) OR placebo*:de,ab,ti OR ((doubl* NEAR/1 blind*):de,ab,ti) OR ((singl* NEAR/1 blind*):de,ab,ti) OR assign*:de,ab,ti OR allocat*:de,ab,ti OR volunteer*:de,ab,ti | | 3949901 |
|  | #7 | (#1 OR #2) AND (#4 AND #5 OR #3) AND #6 | | 644 |
| Web of Science | #1 | TS=(“Ischemic Stroke*” OR “Ischaemic Stroke*” OR “Cryptogenic Embolism Stroke*” OR “Cryptogenic Stroke*” OR “Wake up Stroke*” OR “acute stroke*”) | | 142610 |
|  | #2 | TS=(“Oxygen” OR “oxigen” OR “dioxygen” OR “O2” OR “hyperoxia”) | | 1346861 |
|  | #3 | TS=(“inspir*” OR “inhal*” OR “fraction*” OR “concentrat*” OR “suppl*” OR “therap*” OR “administr*” OR “dosag*” OR “dose*” OR “dosing*” OR “normobaric” OR “eubaric”) | | 11365532 |
|  | #4 | TS= clinical trial* OR TS=research design OR TS=comparative stud* OR TS=evaluation stud* OR TS=controlled trial* OR TS=follow-up stud* OR TS=prospective stud* OR TS=random* OR TS=placebo* OR TS=(single blind*) OR TS=(double blind*) | | 7205331 |
|  | #5 | #1 AND (#2 AND #3) AND #4 | | 650 |
| Cochrane | #1 | MeSH descriptor: [Ischemic Stroke] explode all trees | | 2128 |
|  | #2 | (‘Ischemic Stroke*’ OR ‘Ischaemic Stroke*’ OR ‘Cryptogenic Embolism Stroke*’ OR ‘Cryptogenic Stroke*’ OR ‘Wake up Stroke*’ OR ‘acute stroke*’):ti,ab,kw | | 32534 |
| Cochrane | #3 | MeSH descriptor: [Oxygen] explode all trees | | 7612 |
|  | #4 | MeSH descriptor: [Oxygen Inhalation Therapy] explode all trees | | 2436 |
|  | #5 | MeSH descriptor: [Hyperoxia] explode all trees | | 324 |
|  | #6 | ('oxygen' OR 'oxigen' OR 'dioxygen' OR 'O2' OR 'hyperoxia'): ti,ab,kw | | 75482 |
|  | #7 | ('inspir*' OR 'inhal*' OR 'fraction*' OR 'concentrat*' OR 'suppl*' OR 'therap*' OR 'administr*' OR 'dosag*' OR 'dose*' OR 'dosing*' OR 'normobaric' OR 'eubaric'): ti,ab,kw | | 1402109 |
|  | #8 | (#1 OR #2) AND ( #6 AND #7 OR #3 OR #4 OR #5) | | 845(trials) |
| Clinical  Trials.gov | #1 | ("stroke*" OR "cerebrovascular accident" OR "cerebrovascular accidents") AND ("Oxygen" OR "oxigen" OR "dioxygen" OR "O2" OR "hyperoxia") | | 121 |

2. **Supplemental Figure S1** Quality assessment of included studies using the Cochrane Risk of Bias 2.0 tool.

| 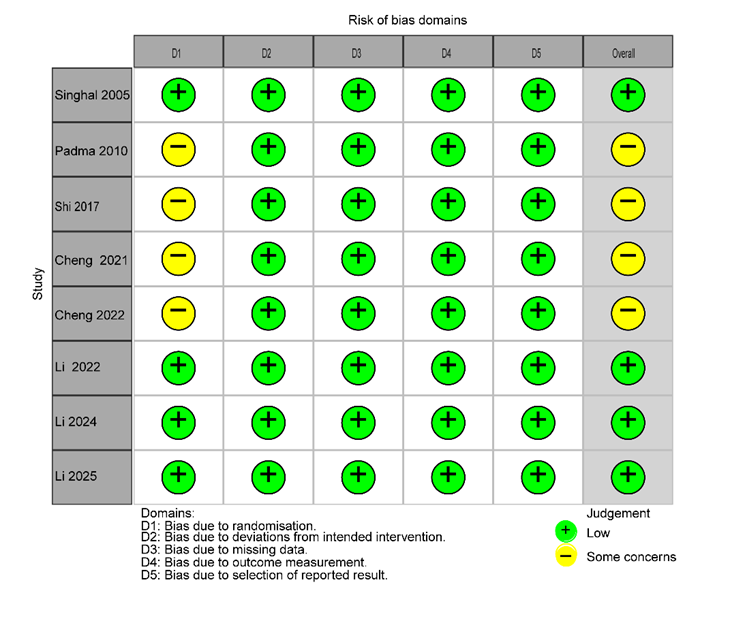 |
| --- |

3. **Supplemental Figure S2** Risk of bias plot over all studies.

| 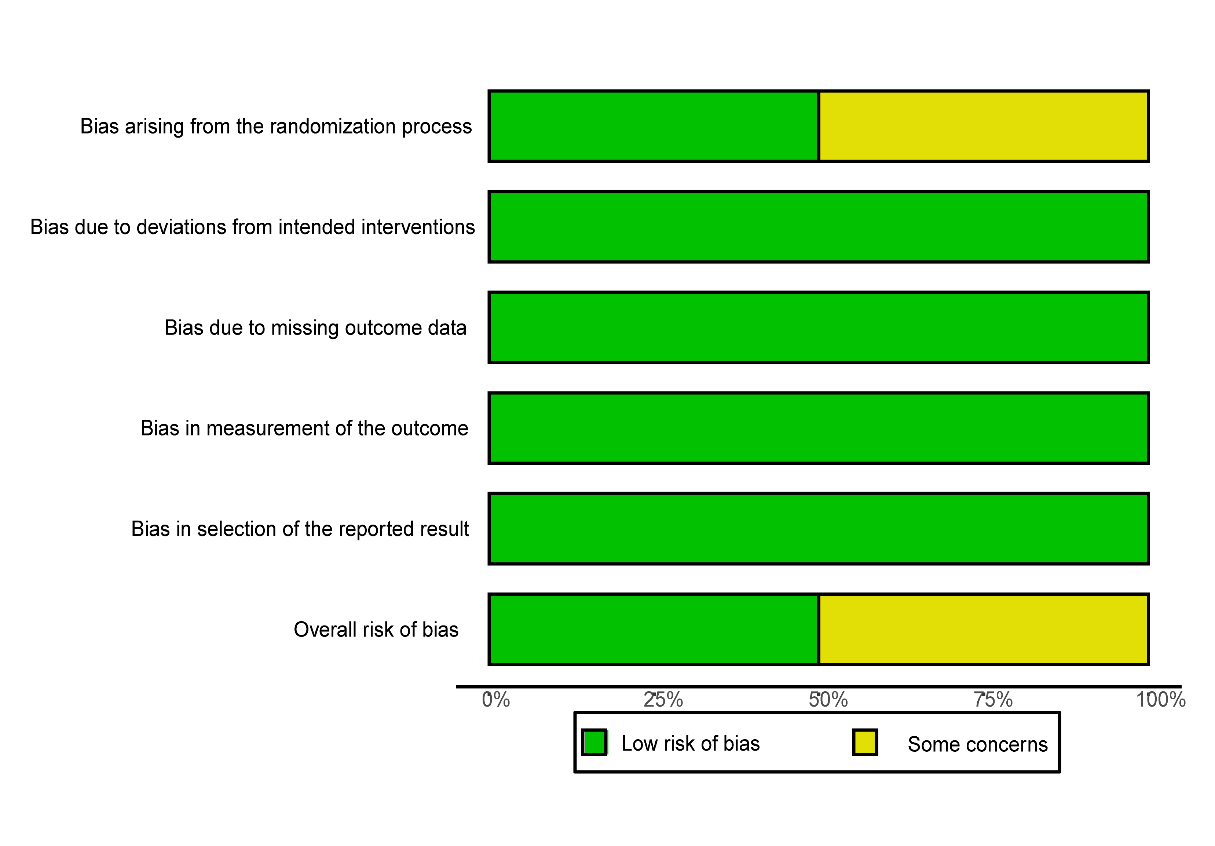 |
| --- |

4. **Supplemental Table S2** GRADE Level of evidence.

| Certainty assessment | | | | | | | Summary of findings | | | |
| --- | --- | --- | --- | --- | --- | --- | --- | --- | --- | --- |
| **Participants (studies) Follow-up** | **Risk of bias** | Inconsistency | Indirectness | Imprecision | Publication bias | Overall certainty of evidence | Study event rates (%) | | Relative effect (95% CI) | Anticipated absolute effects |
|  |  |  |  |  |  |  | With control | With NBHO |  | Risk difference with NBHO |
| Functional independence | | | | | | | | | | |
| 746 (6 RCTs) | serious^a^ | not serious | not serious | serious^b^ | Serious^d^ | ⨁◯◯◯ very Low^a,b,d^ | 157/347 (45.2%) | 229/399 (57.4%) | RR 1.28 (1.07 to 1.51) | 127 more per 1,000 (from 32 more to 231 more) |
| Reduced disability | | | | | | | | | | |
| 746 (6 RCTs) | serious^a^ | not serious | not serious | not serious | serious^d^ | ⨁⨁◯◯ Low^a,d^ | 347 | 399 | cOR 1.72  (1.35 to 2.20) | – |
| Change of NIHSS at 4 hours | | | | | | | | | | |
| 102 (2 RCTs) | serious^a^ | not serious | not serious | serious^b^ | serious^d^ | ⨁◯◯◯  very Low^a,b,d^ | 50 | 52 | – | MD 3.58 lower (13.79 lower to 6.63 higher) |
| Change of NIHSS at 24 hours | | | | | | | | | | |
| 804 (8 RCTs) | serious^a^ | serious^c^ | not serious | not serious | serious^d^ | ⨁◯◯◯ very Low^a,c,d^ | 376 | 428 | – | MD 2.49 lower (5.05 lower to 0.07 higher) |
| Change of NIHSS at 72 hours | | | | | | | | | | |
| 526 (5 RCTs) | serious^a^ | not serious | not serious | serious^b^ | serious^d^ | ⨁◯◯◯ very Low^a,b,d^ | 239 | 287 | – | MD 2.18 lower (3.45 lower to 0.9 lower) |
| Change of NIHSS at 7 days | | | | | | | | | | |
| 542 (6 RCTs) | serious^a^ | not serious | not serious | serious^b^ | serious^d^ | ⨁◯◯◯ very Low^a,b,d^ | 246 | 296 | – | MD 2.23 lower (4.55 lower to 0.1 higher) |
| 90 Day Mortality | | | | | | | | | | |
| 746 (6 RCTs) | serious^a^ | not serious | not serious | serious^b^ | serious^d^ | ⨁◯◯◯ very Low^a,b,d^ | 55/347 (15.9%) | 36/399  (9.0%) | RR 0.62 (0.39 to 0.99) | 64 fewer per 1,000 (from 97 fewer to 2 fewer) |
| Symptomatic intracerebral hemorrhage | | | | | | | | | | |
| 746 (6 RCTs) | serious^a^ | not serious | not serious | serious^b^ | serious^d^ | ⨁◯◯◯ very Low^a,b,d^ | 28/347 (8.1%) | 25/399  (6.3%) | RR 0.79 (0.45 to 1.40) | 17 fewer per 1,000 (from 44 fewer to 32 more) |
| Pneumonia | | | | | | | | | | |
| 644 (4 RCTs) | serious^a^ | not serious | not serious | serious^b^ | serious^d^ | ⨁◯◯◯ very Low^a,b,d^ | 35/297 (11.8%) | 43/347  (12.4%) | RR 0.97 (0.61 to 1.55) | 4 fewer per 1,000 (from 46 fewer to 65 more) |
| CI = confidence interval; MD = mean difference; SD = standard deviation; RR = risk ratio; AIS = acute ischemic stroke; MCID = minimal clinically important difference; NBHO = Normobaric hyperoxia; NIHSS = National Institutes of Health Stroke scale; – = not applicable.  a Downgraded one level for unclear allocation concealment.  b Downgraded one level for imprecision（Small sample size, wide confidence interval, and confidence intervals spanned clinically important threshold (continuous outcomes: MCID =0.5 × total pooled SD and binary outcomes: RR = 1.25);  c Downgraded one level for heterogeneity (I^2^ of 50% to 75%, > 75% considered as medium and large heterogeneity).  d Downgraded one level for inability to assess publication bias (with only 8 studies included)  GRADE Working Group grades of evidence.  High quality: further research is very unlikely to change our confidence in the estimate of effect.  Moderate quality: further research is likely to have an important impact on our confidence in the estimate of effect and may change the estimate.  Low quality: further research is very likely to have an important impact on our confidence in the estimate of effect and is likely to change the estimate.  Very low quality: we are very uncertain about the estimate. | | | | | | | | | | |

5. **Supplemental Figure S3** Forest plot of functional outcomes by secondary model:(A) independence (mRS 0-2 at 90 days) and (B) reduced disability (ordinal shift across mRS grades 0-6 at 90 days).

| 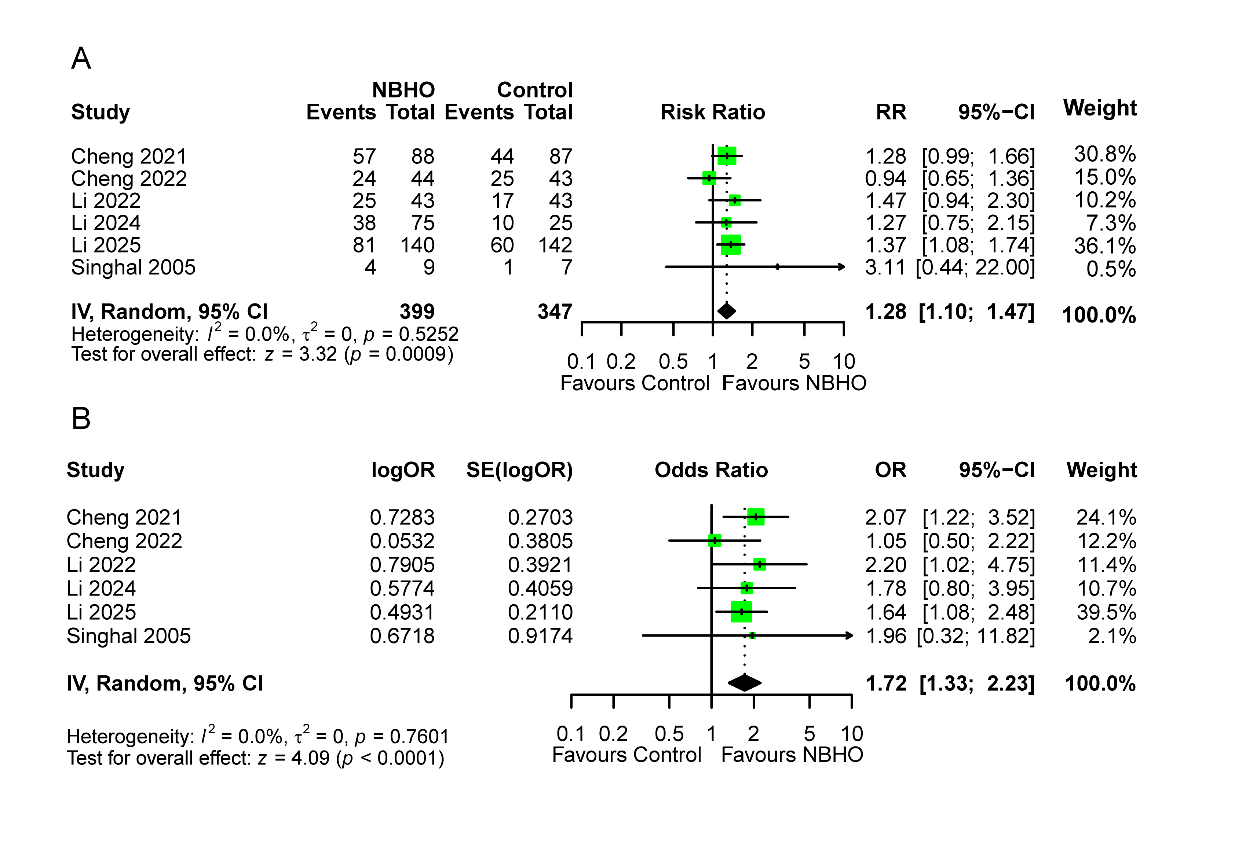 |
| --- |

Note: secondary model = random-effects model without Hartung-Knapp-Sidik-Jonkman adjustment. The model specifications above apply uniformly to all forest plots

6.**Supplemental Figure S4** Sensitivity analysis of functional outcomes by primary model: (A) independence (mRS 0-2 at 90 days) and (B) reduced disability (ordinal shift across mRS grades 0-6 at 90 days).

| 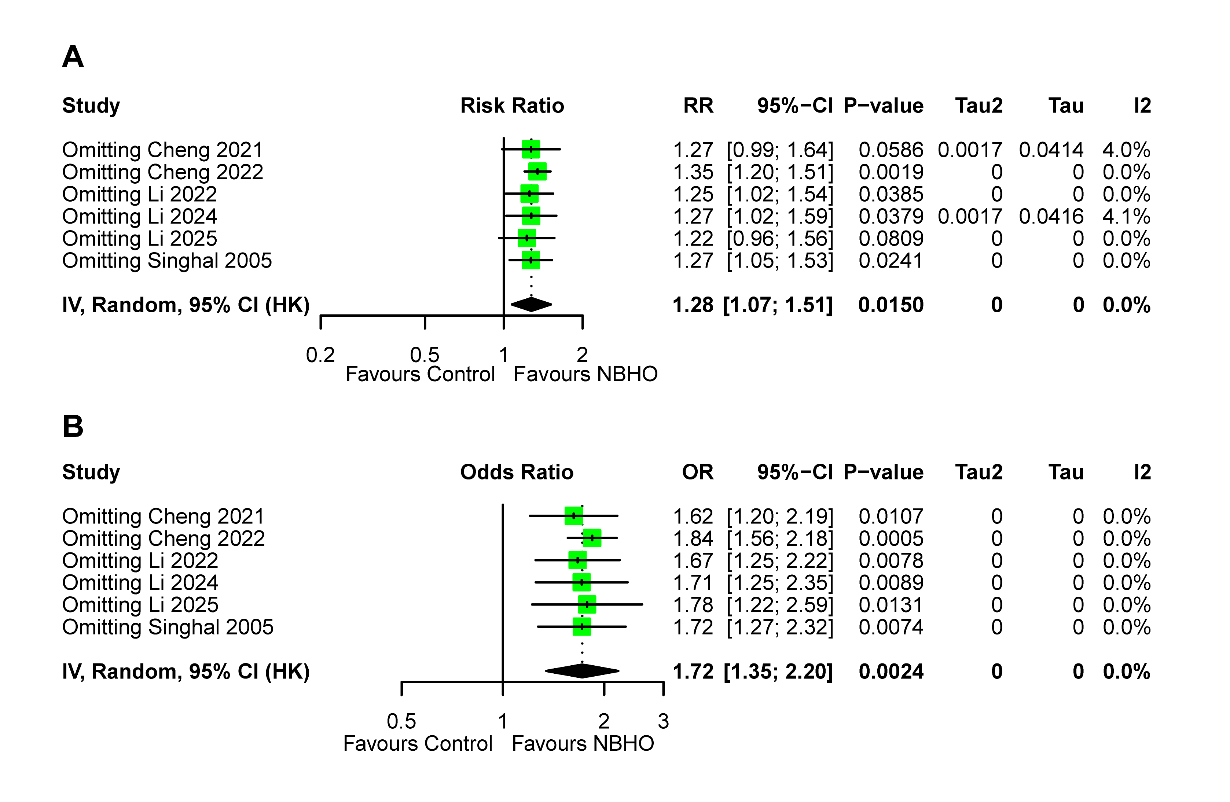 |
| --- |

7.**Supplemental Figure S5** Sensitivity analysis of functional outcomes by secondary model: (A) independence (mRS 0-2 at 90 days) and (B) reduced disability (ordinal shift across mRS grades 0-6 at 90 days).

| 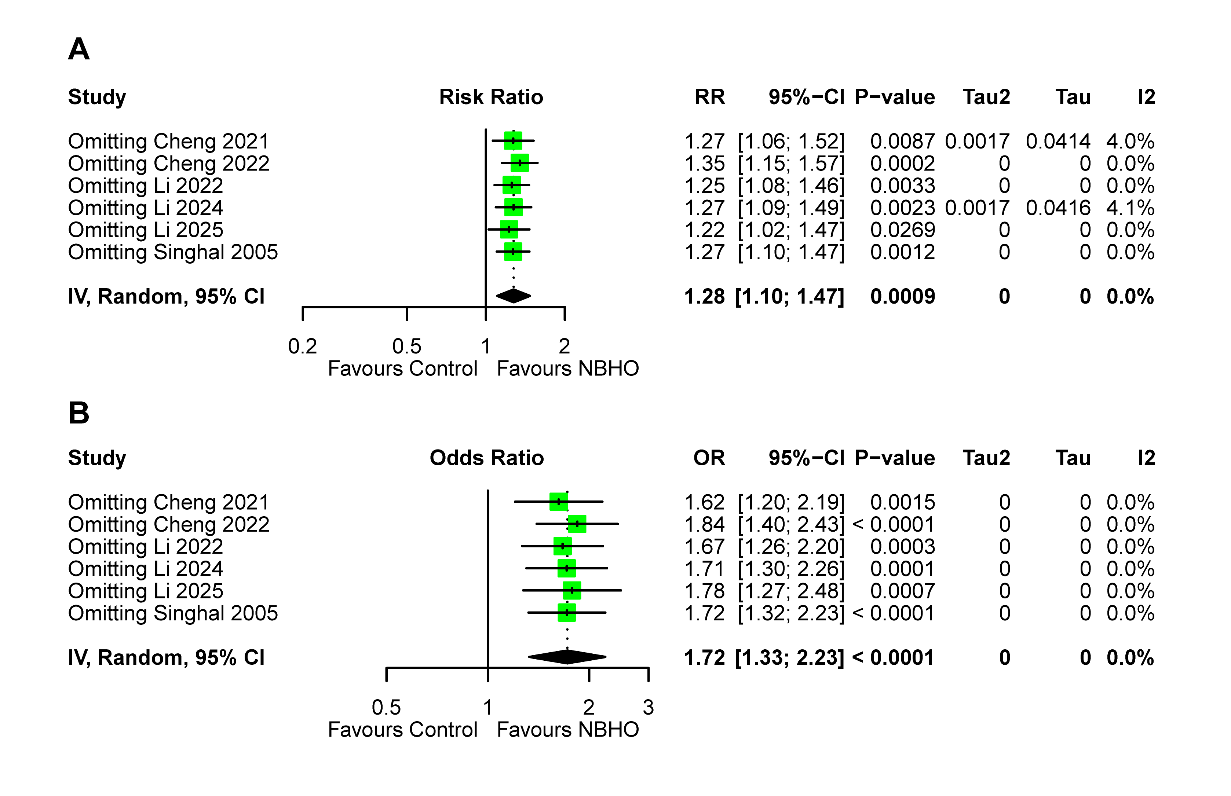 |
| --- |

8. **Supplemental Figure S6** Forest plots of subgroup analysis for functional outcomes by primary model: (A) independence (mRS 0-2 at 90 days) in anterior circulation stroke subgroup, (B) independence (mRS 0-2 at 90 days) in EVT subgroup (C) reduced disability (ordinal shift across mRS grades 0-6 at 90 days) in anterior circulation stroke subgroup, and (D) reduced disability (ordinal shift across mRS grades 0-6 at 90 days) in EVT subgroup.

| 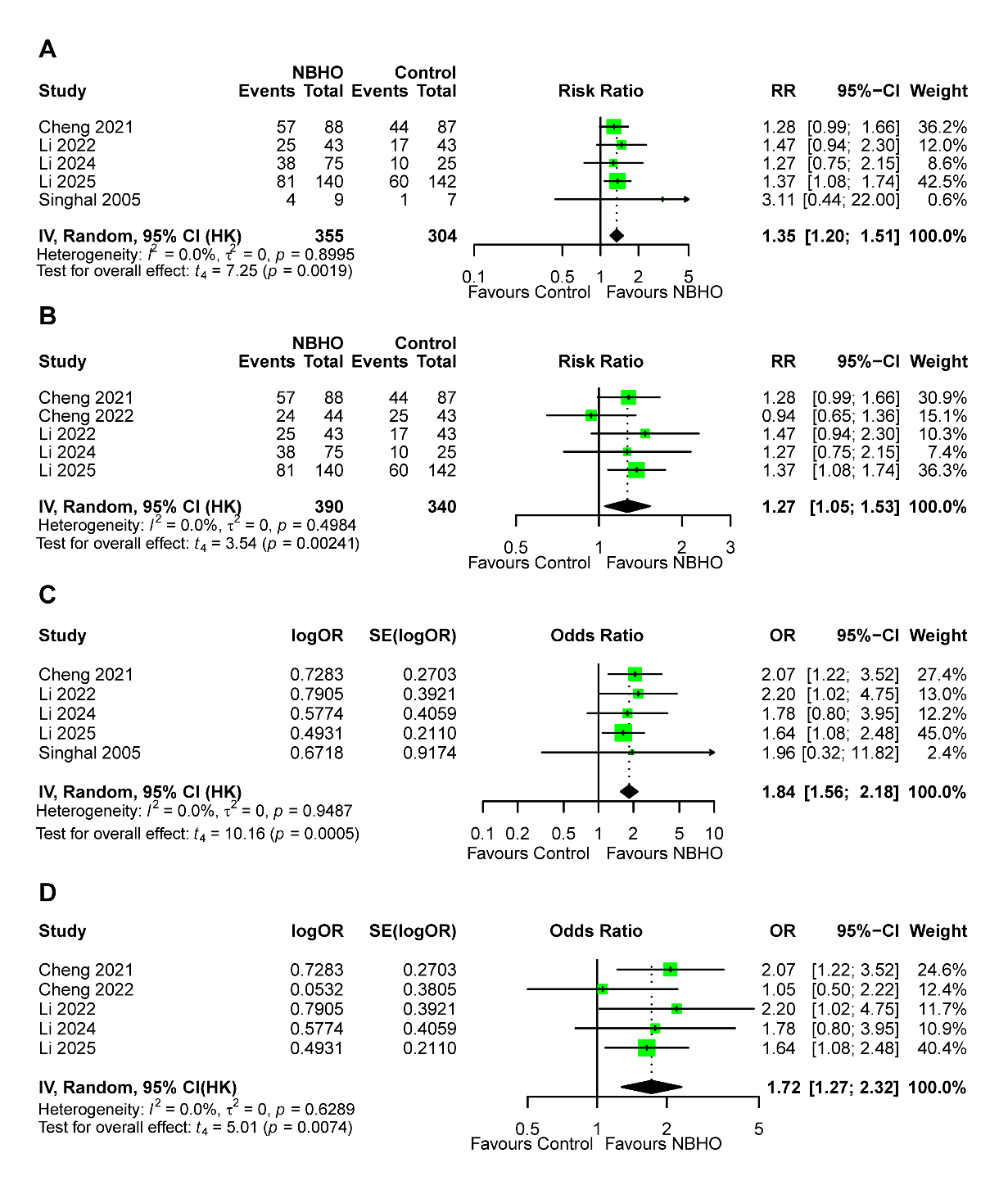 |
| --- |

9. **Supplemental Figure S7** Forest plots of subgroup analysis for functional outcomes by secondary model: (A) independence (mRS 0-2 at 90 days) in anterior circulation stroke subgroup, (B) independence (mRS 0-2 at 90 days) in EVT subgroup (C) reduced disability (ordinal shift across mRS grades 0-6 at 90 days) in anterior circulation stroke subgroup, and (D) reduced disability (ordinal shift across mRS grades 0-6 at 90 days) in EVT subgroup.

| 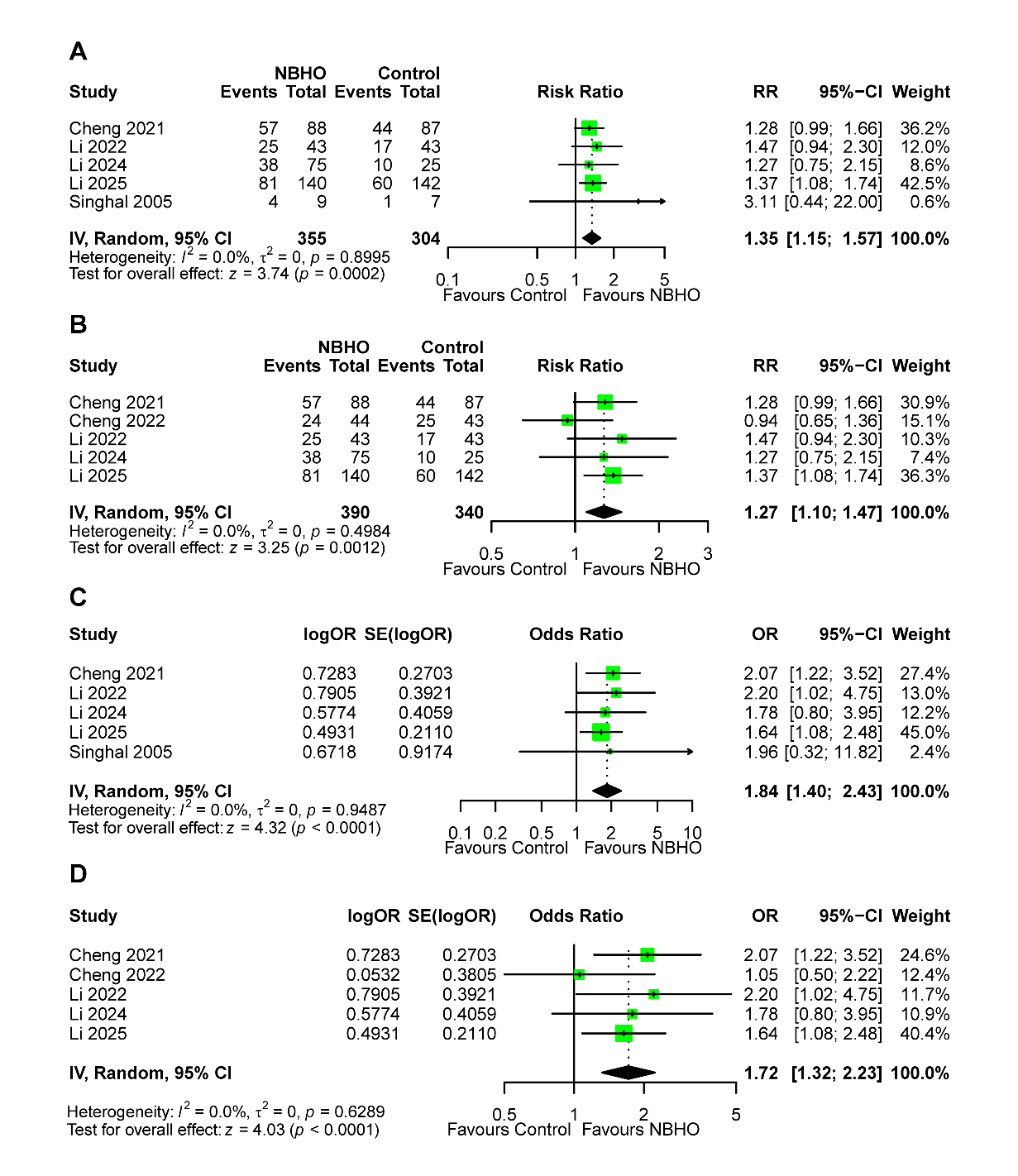 |
| --- |

10. **Supplemental Figure S8** Forest plots of NIHSS scores changes by primary model: at 24 hours in anterior subgroup.

| 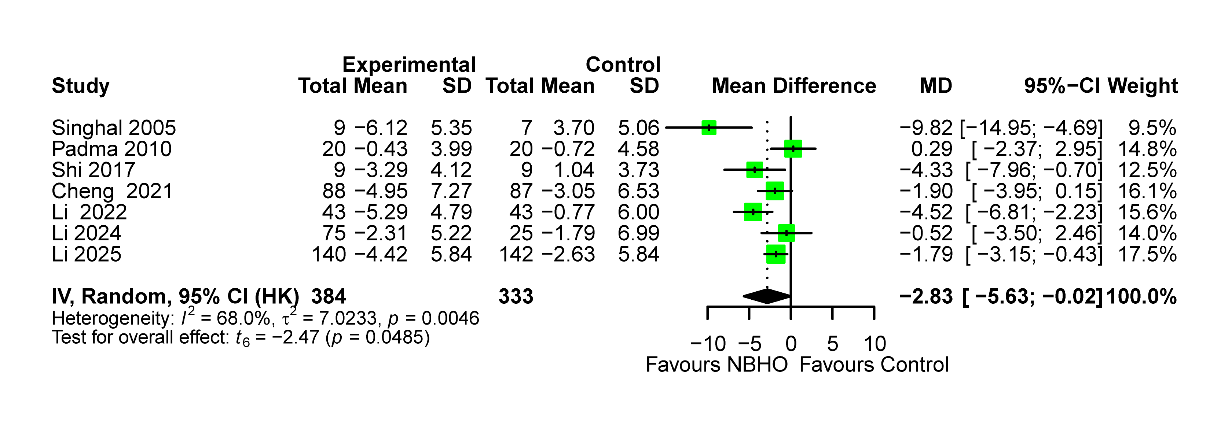 |
| --- |

11. **Supplemental Figure S9** Forest plots of efficacy outcomes by secondary model: (A) NIHSS scores changes at 4 hours and (B) NIHSS scores changes at 24 hours in anterior subgroup.

| 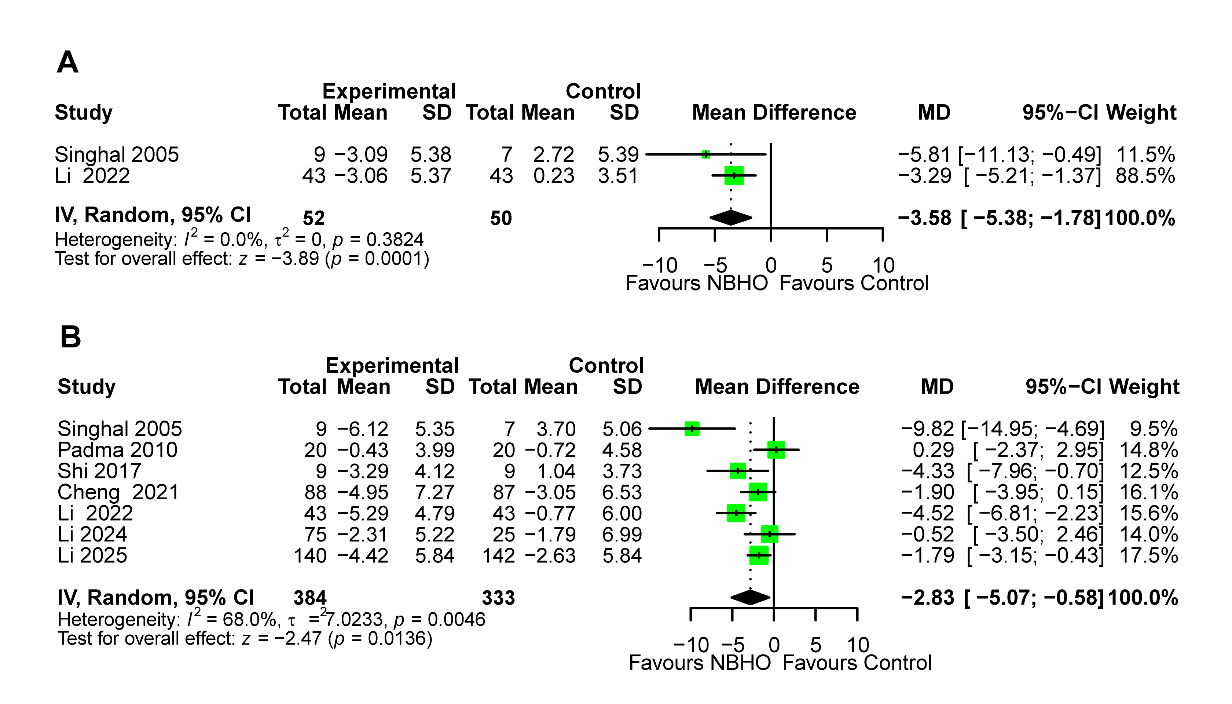 |
| --- |

12. **Supplemental Figure S10** Forest plots of NIHSS scores changes by secondary model: (A) 24 hours, (B) 72 hours, and (C) 7 days

| 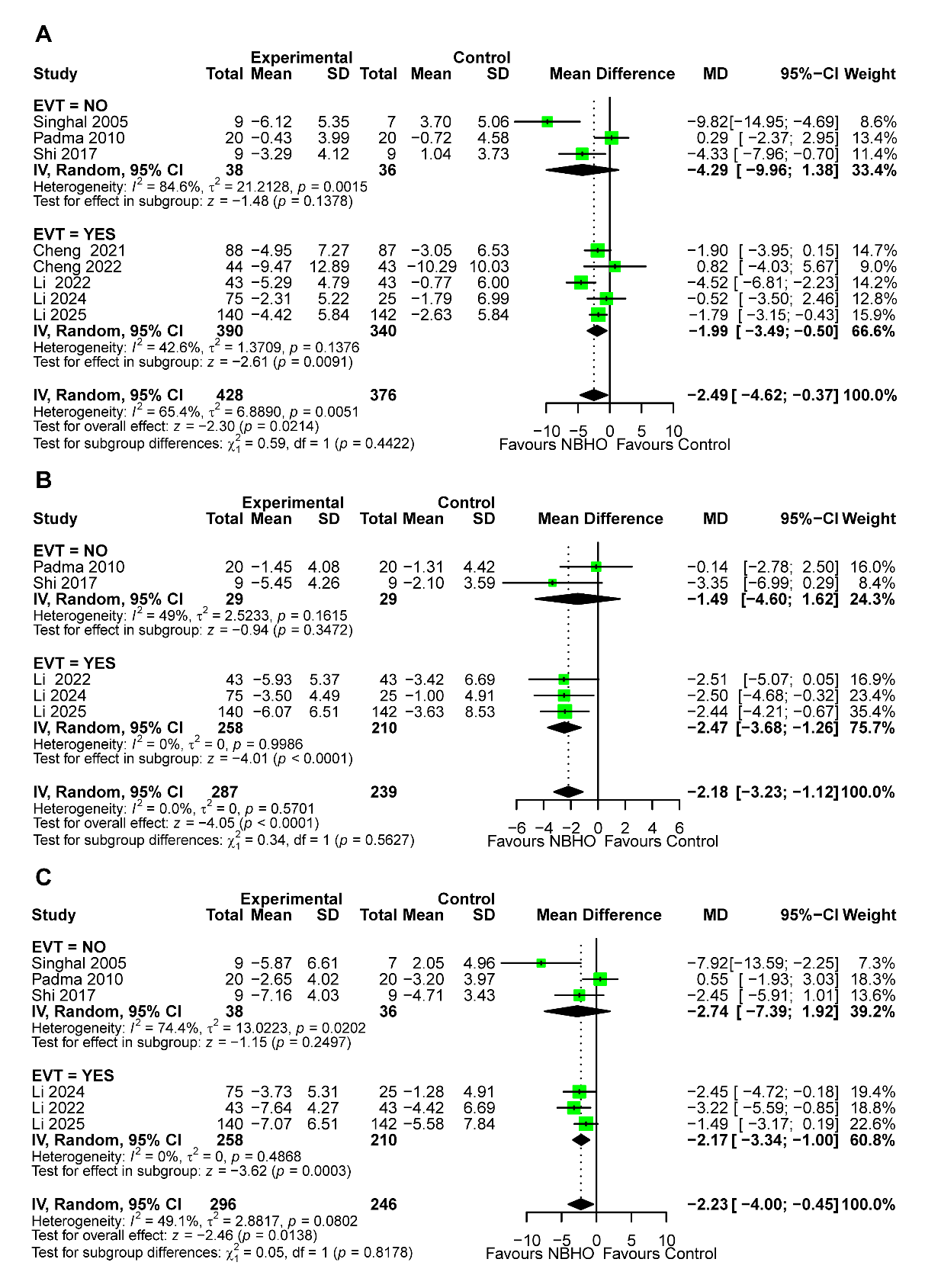 |
| --- |

13. **Supplemental Figure S11** Sensitivity analysis of NIHSS scores changes by primary model: (A) 24 hours, (B) 72 hours, and (C) 7 days.

| 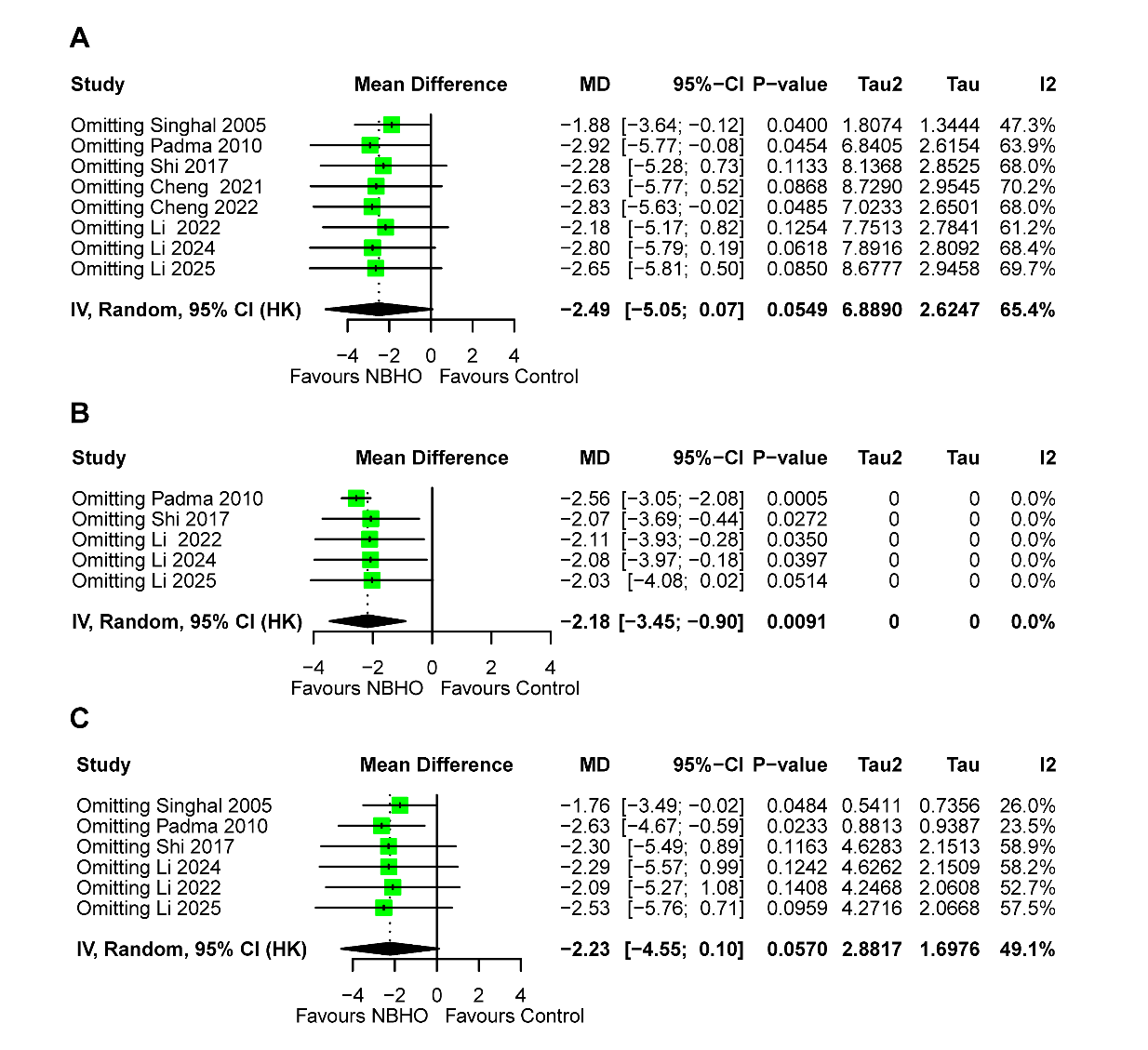 |
| --- |

14. **Supplemental Figure S12** Sensitivity analysis of NIHSS scores changes by secondary model: (A) 24 hours, (B) 72 hours, and (C) 7 days.

| 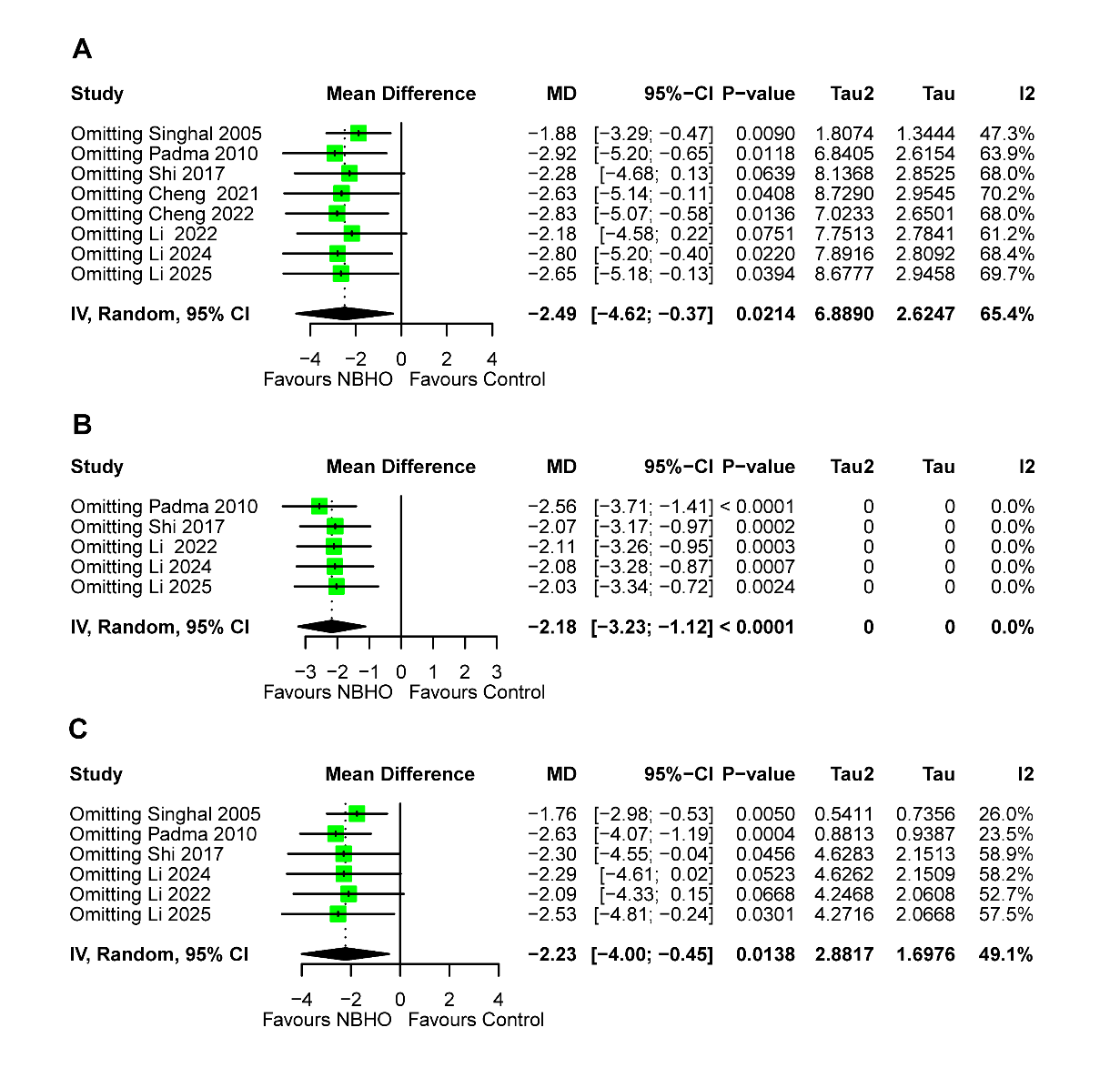 |
| --- |

15. **Supplemental Table S3** Infarct volume across studies and time points.

| **Study** | **Infarct volume** ^a^ **Measurement Time** | **NBHO group (ml)** | **Control group (ml)** |
| --- | --- | --- | --- |
| Singhal 2005 | 4 h | 4 (2.6–4.7)^*^ | 4.5 (3.5–5.7) |
|  | 24 h | 24.4 (21.3–26.5) | 25 (22.5–27.7) |
| Padma 2010^b^ | 24 h | 96.4 | 94.1 |
| Cheng 2021 | 24 h | 9.4 (4.0–35)^*^ | 20.5 (6.45–111) |
| Cheng 2022 | 24 h | 6.5 (2.0–18.0) | 6.75 (3.0–34.5) |
| Li 2022 | 24–48 h | 20.1 (4.3–40.3)^*^ | 37.7 (14.4–124.2) |
| Li 2024 (three subgroup^c^) | | | |
| NBHO:2 h (10 L/min) | 72 h | 30.6 ± 30.1 | 39.4 ± 34.3 |
| NBHO:4 h (10 L/min) |  | 19.7 ± 15.4^*^ |  |
| NBHO:6 h (10 L/min) |  | 22.6 ± 22.4^*^ |  |
| Li 2025 | 24–48 h | 19 (9–37)^*^ | 27 (12–78) |

**^a^** Infarct volume data format: mean ± SD or median (IQR).

^*^ *P*<0.05, compared with control group.

^b^ Padma 2010 reported mean value only.

^c^ Three NBHO subgroup with oxygen 10 L/min for 2 h, 4 h and 6 h respectively.

Abbreviations: NBHO = normobaric hyperoxia; IQR = interquartile range; SD = standard deviation

16. **Supplemental Figure S13** Forest plots of safety outcomes by secondary model: (A) 90-day mortality, (B) 24-hour sICH, and (C) pneumonia.

| 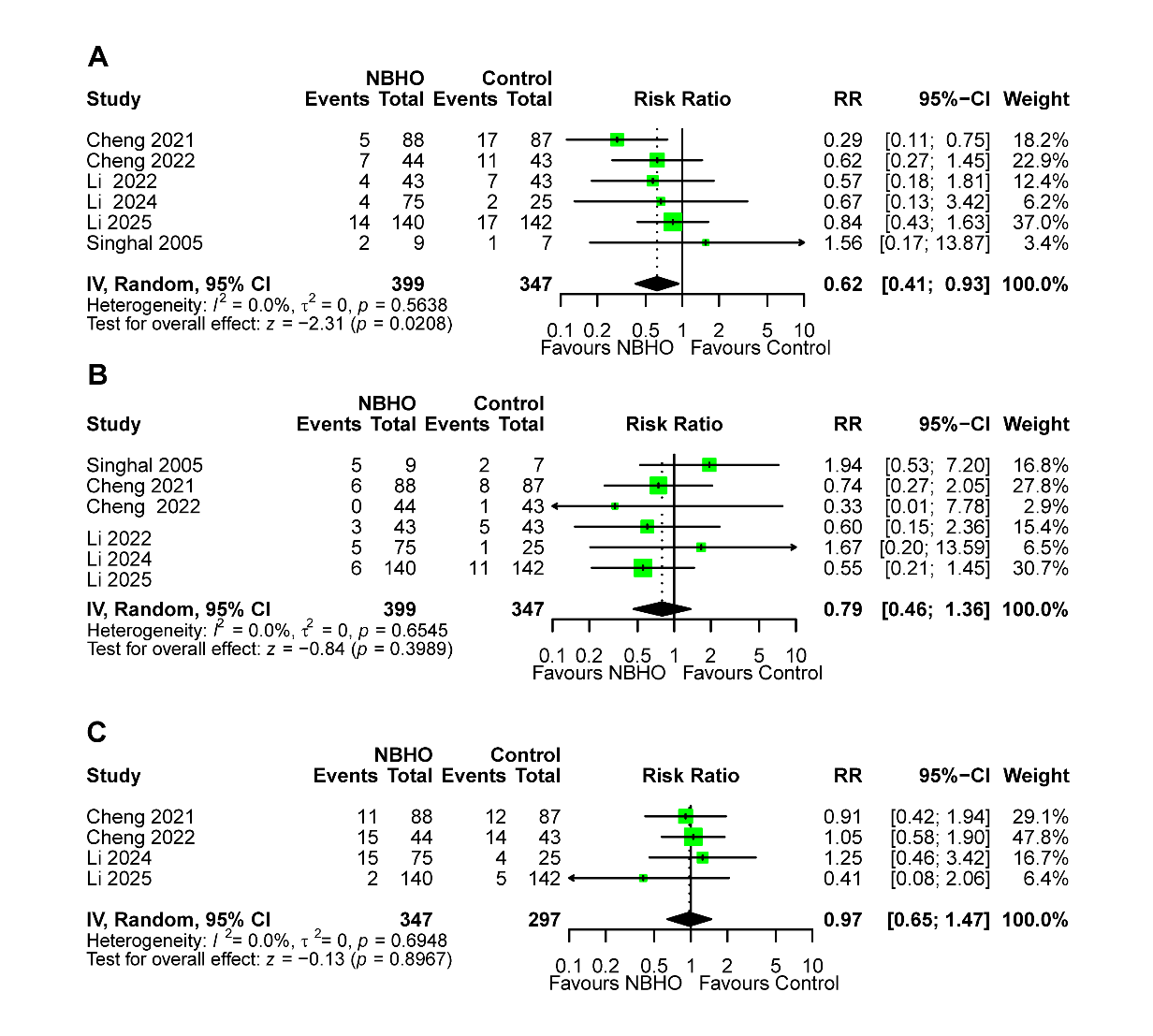 |
| --- |

17.**Supplemental Figure S14** Sensitivity analysis of safety outcomes by primary model: (A) 90-day mortality, (B) 24-hour sICH, and (C) pneumonia.

| **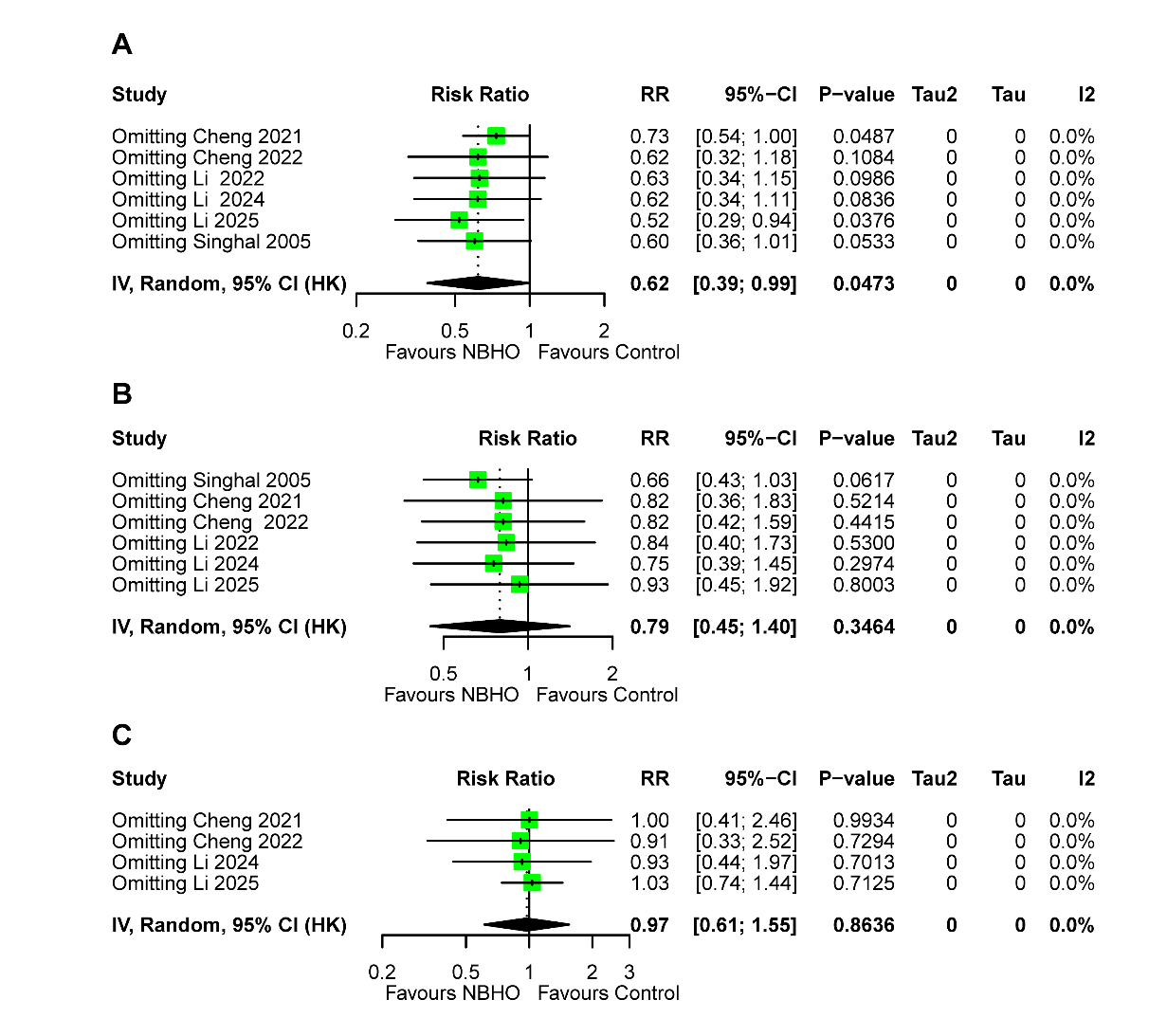** |
| --- |

18.**Supplemental Figure S15** Sensitivity analysis of safety outcomes by secondary model: (A) 90-day mortality, (B) 24-hour sICH, and (C) pneumonia.

| 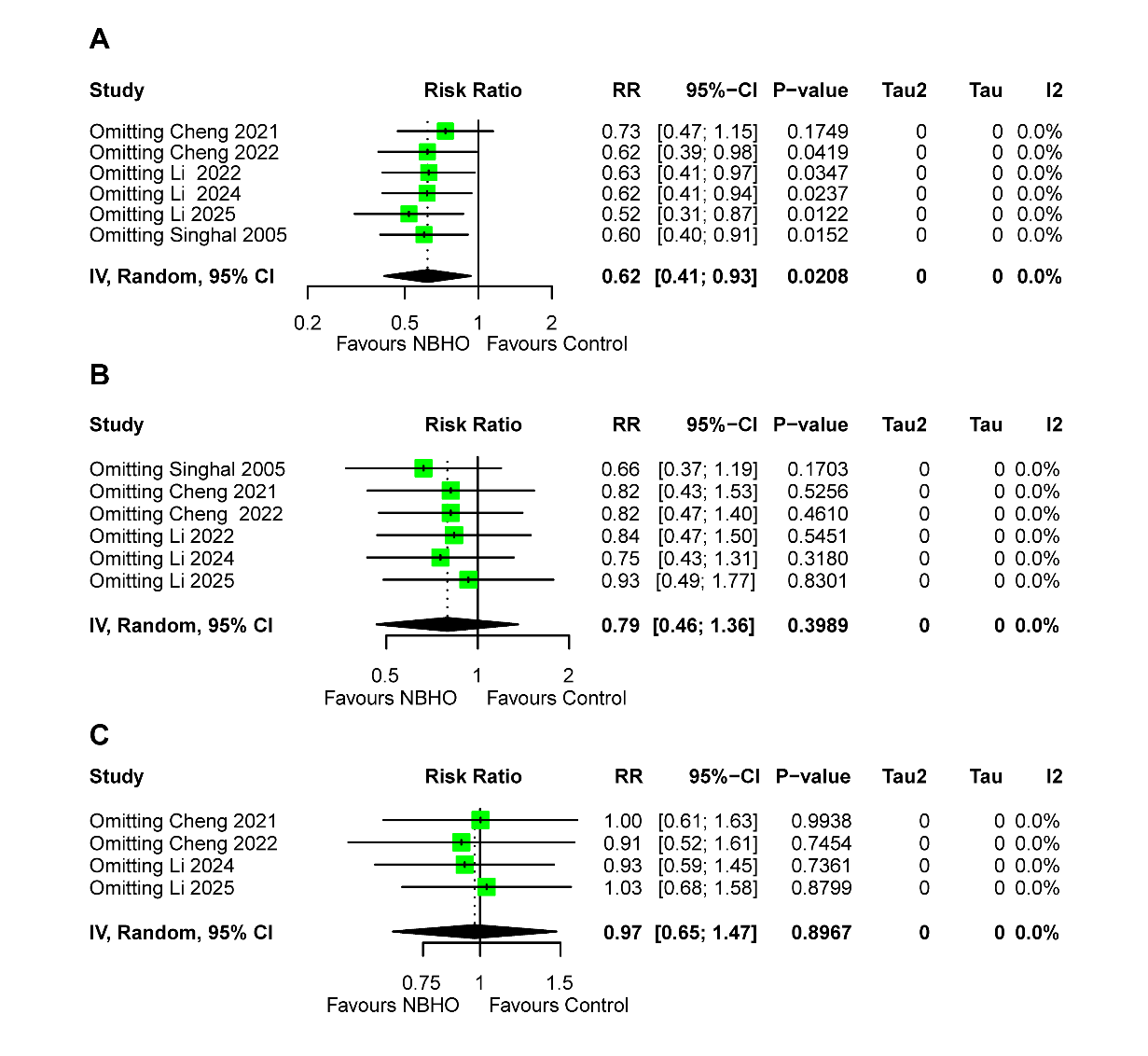 |
| --- |

19. **Supplemental Figure S16** Forest plots of subgroup analysis for safety outcomes by primary model: (A) 90-day mortality in anterior subgroup, (B) 90-day mortality in EVT subgroup, (C) 24-hour sICH in anterior subgroup, (D) 24-hour sICH in EVT subgroup, and (E) pneumonia in anterior subgroup.

| 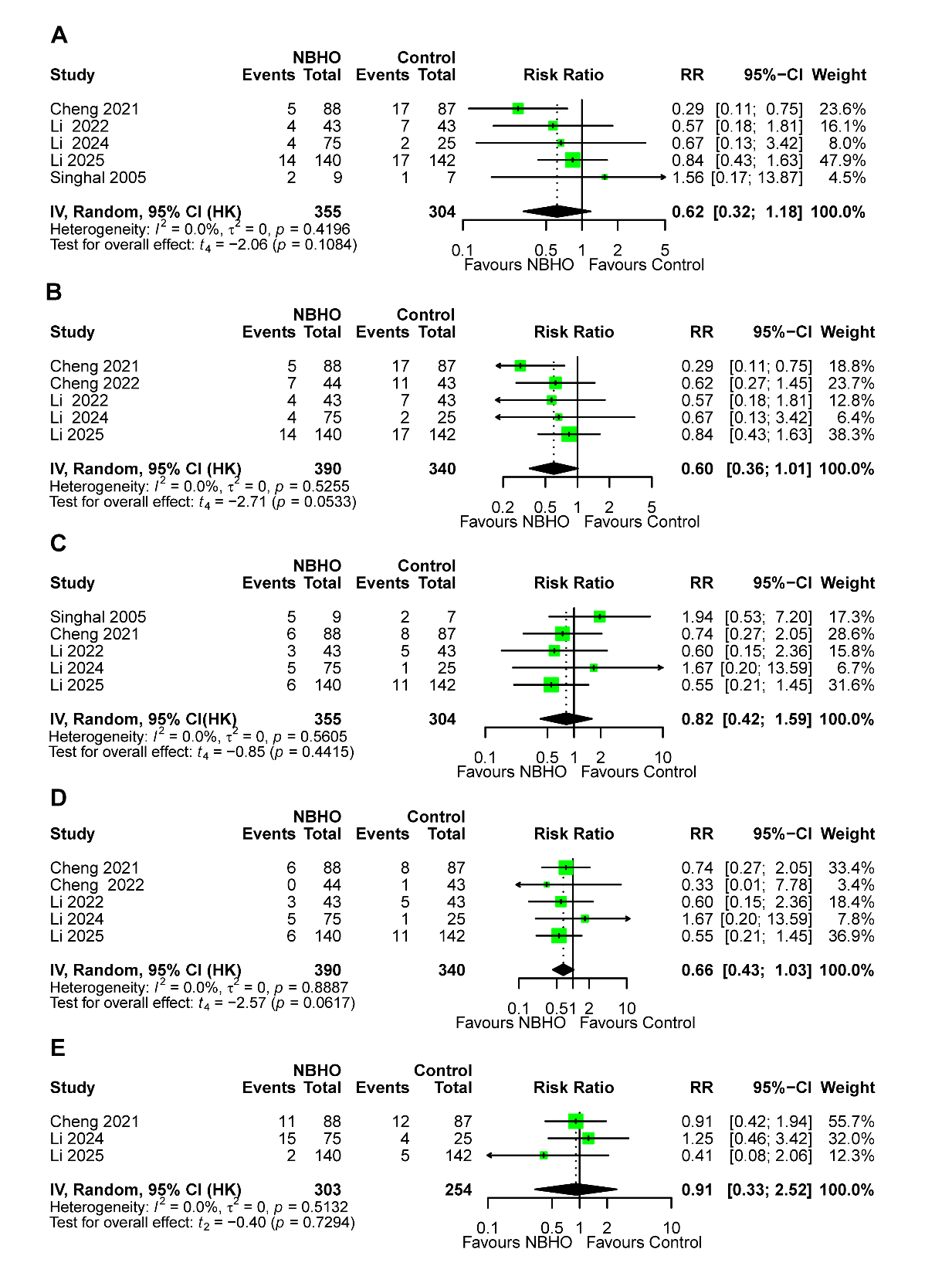 |
| --- |

20.**Supplemental Figure S17** Forest plots of subgroup analysis for safety outcomes by secondary model: (A) 90-day mortality in anterior subgroup, (B) 90-day mortality in EVT subgroup, (C) 24-hour sICH in anterior subgroup, (D) 24-hour sICH in EVT subgroup, and (E) pneumonia in anterior subgroup.

| 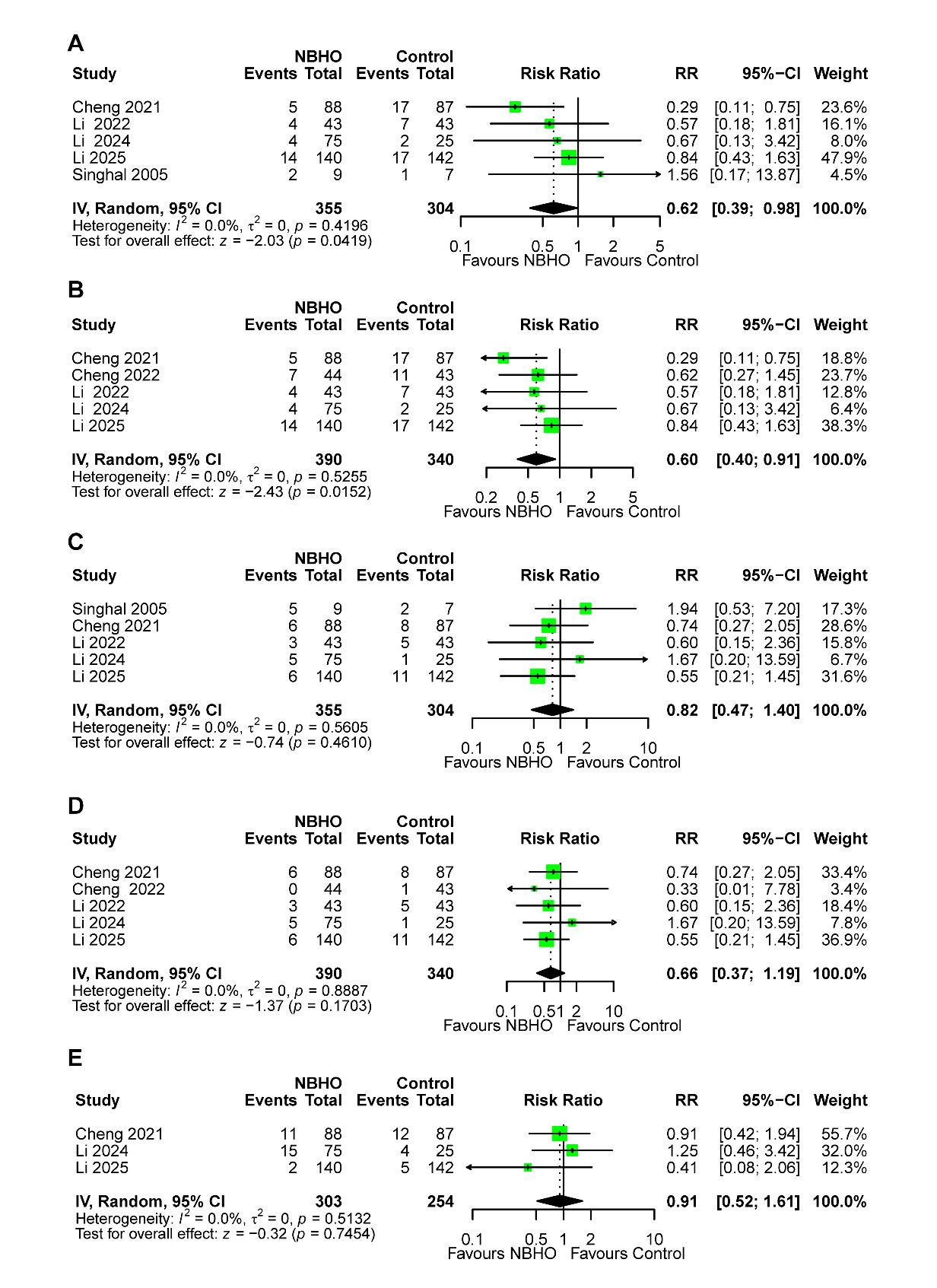 |
| --- |
