# Supplementary material for: Efficacy and safety of normobaric hyperoxia for acute ischemic stroke: a systematic review and meta-analysis of randomized controlled trials
Source: eClinicalMedicine. 2025 Dec 18;91:103701. doi: 10.1016/j.eclinm.2025.103701 (PMC12775875; doi:10.1016/j.eclinm.2025.103701)
Supplement: Supplementary File 1 [file mmc2.docx]

**Content**

**Supplementary file 1 Table 1.** Risk of bias assessment for the Singhal 2010 trial using the RoB 2.0 tool.

**Supplementary file 1 Figure 1** Forest plots of efficacy outcomes in the analysis including Singhal 2010 : (A) NIHSS scores changes at 4 hours and (B) NIHSS scores changes at 24 hours.

**Supplementary file 1 Figure 2** 90-day mortality in the analysis including Singhal 2010.

**Supplementary file 1 Table 1.** Risk of bias assessment for the Singhal 2010 trial using the RoB 2.0 tool.

| Study | Randomization process | Deviations from intended interventio | Missing outcome data | Measurement of the outcome | Selection of the reported result | Overall bias |
| --- | --- | --- | --- | --- | --- | --- |
| Singhal 2010 | High | low | High | low | High | High |

**Supplementary file 1 Figure 1** Forest plots of efficacy outcomes in the analysis including Singhal 2010 : (A) NIHSS scores changes at 4 hours and (B) NIHSS scores changes at 24 hours.

| 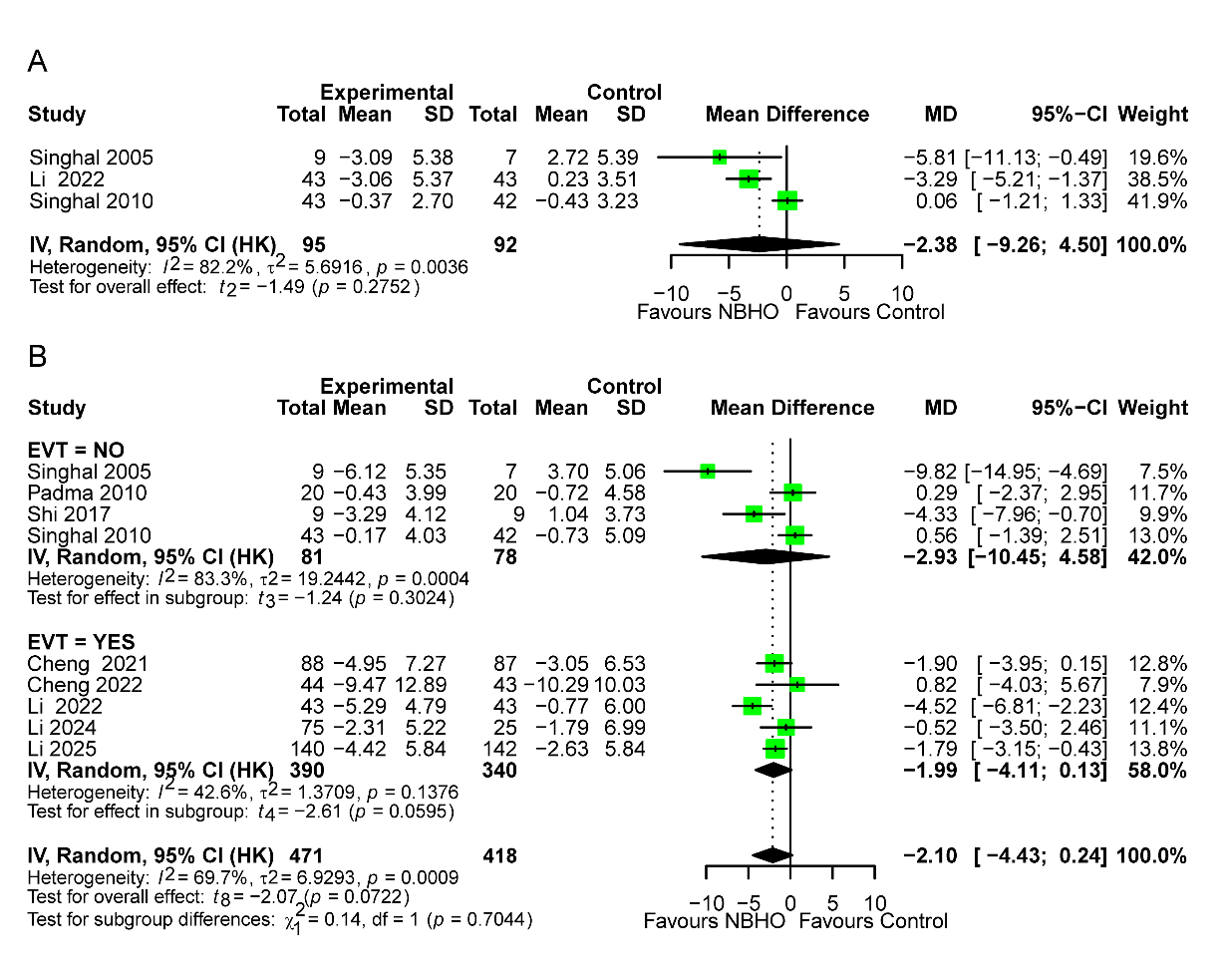 |
| --- |

**Supplementary file 1 Figure 2** 90-day mortality in the analysis including Singhal 2010.

| 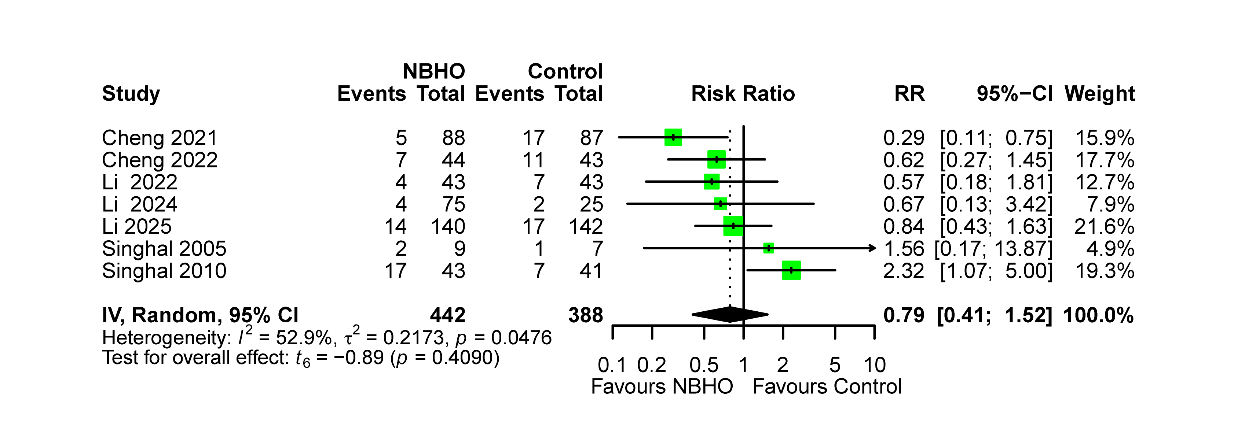 |
| --- |
